# The role of radiotherapy-related autophagy genes in the prognosis and immune infiltration in lung adenocarcinoma

**DOI:** 10.3389/fimmu.2022.992626

**Published:** 2022-10-13

**Authors:** Jingyan Gao, Fei Lu, Jiawen Yan, Run Wang, Yaoxiong Xia, Li Wang, Lan Li, Li Chang, Wenhui Li

**Affiliations:** ^1^ Department of Radiation Oncology, The Third Affiliated Hospital of Kunming Medical University, Tumor Hospital of Yunnan Province, Kunming, China; ^2^ Department of Oncology and Hematology, Southern Central Hospital of Yunnan Province, The First People’s Hospital of Honghe State, Mengzi, China

**Keywords:** lung adenocarcinoma, radiotherapy, prognosis, autophagy, tumor immune microenvironment

## Abstract

**Background:**

There is a close relationship between radiotherapy and autophagy in tumors, but the prognostic role of radiotherapy-related autophagy genes (RRAGs) in lung adenocarcinoma (LUAD) remains unclear.

**Methods:**

Data used in the current study were extracted from The Cancer Genome Atlas (TCGA) and Gene Expression Omnibus (GEO) databases. Weighted gene co-expression network analysis (WGCNA) was executed to recognize module genes associated with radiotherapy. The differentially expressed genes (DEGs) between different radiotherapy response groups were filtered *via* edgeR package. The differentially expressed radiotherapy-related autophagy genes (DERRAGs) were obtained by overlapping the module genes, DEGs, and autophagy genes (ATGs). Then, prognostic autophagy genes were selected by Cox analyses, and a risk model and nomogram were subsequently built. Gene Set Enrichment Analysis (GSEA) and single-sample Gene Set Enrichment Analysis (ssGSEA) were performed to investigate potential mechanisms through which prognostic autophagy signatures regulate LUAD. Radiotherapy-resistant cell lines (A549IR and PC9IR) were established after exposure to hypo-fractionated irradiation. Ultimately, mRNA expression was validated by quantitative real-time PCR (qRT-PCR), and relative protein levels were measured in different cell lines by western blot.

**Results:**

A total of 11 DERRAGs were identified in LUAD. After Cox analyses, *SHC1*, *NAPSA*, and *AURKA* were filtered as prognostic signatures in LUAD. Then, the risk score model was constructed using the prognostic signatures, which had a good performance in predicting the prognosis, as evidenced by receiver operating characteristics curves. Furthermore, Cox regression analyses demonstrated that risk score was deemed as an independent prognostic factor in LUAD. Moreover, GSEA and ssGSEA results revealed that prognostic RRAGs may regulate LUAD by modulating the immune microenvironment and affecting cell proliferation. The colony formation assay showed that the radiosensitivity of radiation-resistant cell lines was lower than that of primary cells. The western blot assay found that the levels of autophagy were elevated in the radiotherapy-resistant cell lines. Moreover, the expression of DERRAGs (*SHC1*, *AURKA*) was higher in the radiotherapy-resistant cells than in primary cells.

**Conclusion:**

Our study explored the role of RRAGs in the prognosis of LUAD and identified three biomarkers. The findings enhanced the understanding of the relationship between radiotherapy, autophagy, and prognosis in LUAD and provided potential therapeutic targets for LUAD patients.

## Introduction

Lung cancer is the second leading cause of cancer death after breast cancer worldwide (1). Lung adenocarcinoma (LUAD) is the most common histological subtype of lung cancer (2). Although radiation therapy is the standard therapy for lung cancer patients, the effect of radiation therapy varies, with different responses in patients, especially in LUAD. Many LUAD patients display increased radiation resistance. Radiation resistance is a major cause of LUAD therapeutic failure, resulting in tumor recurrence and metastasis (3). Consequently, research on the mechanism of radiation resistance and the discovery of related biological markers for optimal lung cancer radiation therapy remains an urgent task.

Autophagy is one of the main metabolic pathways that degrade long-lived proteins, senescent organelles, and parts of the cytoplasm in cells (4). Autophagy can influence the effect of radiation therapy by modulating the sensitivity of radiotherapy and altering the immune microenvironment of tumors after radiation. The intricate relationship that exists between cellular autophagy and cell death can both enable cells to survive in adverse environments and promote autophagic cell death. However, new evidence suggests that autophagy is a cytoprotective response that assists tumor cells to cope with survival stress and thus causes resistance to radiotherapy (5, 6). For instance, the upregulation of lncRNA KCNQ1OT1 induces radiotherapy resistance, which is directly attributable to ATGs-regulated autophagy in LUAD cells (7). Therefore, targeting the autophagy-related genes (ARGs) provides new insights into the mechanism of radiotherapy resistance in LUAD and is also significant for boosting the efficacy of radiotherapy in LUAD patients. However, so far, the effects of ARGs on radiotherapy in LUAD patients remain to be further explored. In this study, we sought to determine prognostic RRAGs and elucidate their potential molecular mechanisms in LUAD.

## Materials and methods

### Study design

**Figure 1:**
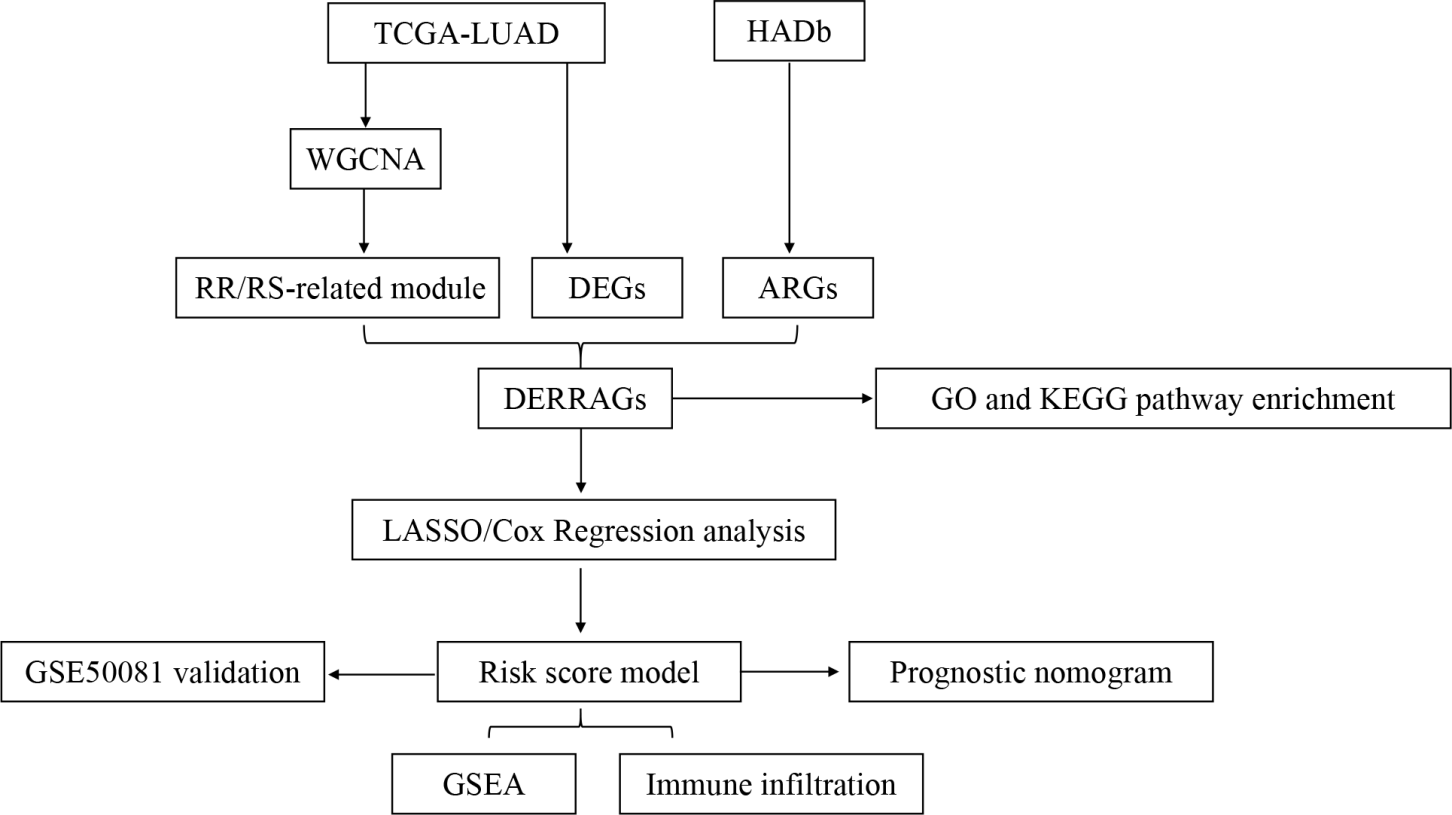
Flowchart of the comprehensive analysis process in the present study.

The flowchart of our study is depicted in **Figure 1**.

### Data source

Gene expression profile and clinical data of LUAD cases were extracted from The Cancer Genome Atlas (TCGA) (https://portal.gdc.cancer.gov/) and Gene Expression Omnibus (GEO) (https://www.ncbi.nlm.nih.gov/geo/) databases. In TCGA-LUAD cohort, there were 497 LUAD and 54 control samples. Among the 497 LUAD samples, 115 samples from patients with complete or partial response were defined as the radiosensitive (RS) group, and 14 samples from patients with progressive disease or stable disease were defined as the radioresistance (RR) group. Moreover, 127 LUAD samples from the GSE50081 dataset were used as an external validation set, and 1,183 autophagy genes (ATGs) (**Table S1**) were downloaded from the Autophagy database (http://www.tanpaku.org/autophagy/).

### WGCNA analysis

The samples were initially clustered to check the overall correlation of all the samples, and the outlier samples were excluded to ensure the accuracy of the analysis. Based on the gene expressions in the RS and RR groups, WGCNA was performed. Then, we determined the optimal soft threshold of the data to ensure that the genes interaction conformed to the scale-free distribution to a maximum extent. The adjacency and similarity between genes were calculated, and the cluster dendrogram was established. The modules were further segmented using the dynamic tree-cutting algorithm, and similar modules were merged. We evaluated the Pearson correlation between each module and sample traits and screened out the module genes with the highest absolute value for the downstream analysis.

### Identification and function analysis of DERRAGs involved in LUAD

DEGs between the RR and RS groups were identified using “edgeR” package (version 3.34.1), with threshold of p < 0.05 and |log2FC| > 0.5, and “ggplot2” package (version 3.3.5) was used to generate the volcano plot to show the DEGs. The expressions of the top 100 DEGs were displayed in the heatmap generated by “pheatmap” package (version 1.0.12). Then, DERRAGs were obtained by overlapping DEGs with module genes and ATGs. “clusterprofiler” package (version 4.0.2) was applied to analyze the function of DERRAGs. The pathways with p < 0.05 were considered as markedly enriched GO terms and KEGG pathways.

### Construction and validation of the risk score model

The risk model was established using 381 LUAD samples with survival information in TCGA-LUAD cohort. These samples were randomly split into training set (n = 267) and internal validation set (n = 114) in a ratio of 7:3. We first implemented univariate Cox regression to filter DERRAGs markedly relevant to overall survival (OS) (p < 0.05) using “survminer” package (version 0.4.8). Thereafter, multivariate Cox regression was applied to build the risk score model, and the risk score was calculated using the formula:


h0(t)*exp(β1X1+β2X2+…+βnXn)

In this formula, β refers to the coefficient, for which the Hazard Ratio (HR) value can be obtained after taking the inverse natural exp(β). LUAD cases in the TCGA training set were split into low- and high-risk groups based on the median risk score. Kaplan–Meier method was used to assess the OS, and the receiver operating characteristic (ROC) curves were plotted using the “survivalROC” (version 1.0.3) package with survival time points of 1-5 years. The risk model was examined in both the TCGA and GEO validation sets. Subsequently, the independent prognostic factors for LUAD were confirmed by univariate and multivariate Cox analyses. The 1-, 3-, and 5-year survival in LUAD cases was predicted through a nomogram, and the calibration curve was utilized to assess the performance of the nomogram.

### GSEA analysis

To further probe the related signaling pathways of the prognostic gene, “clusterprofiler” package (version 3.18.1) and org.Hs.eg.db (version 3.12.0) were used to perform GSEA analysis. The reference gene sets “c2.cp.kegg.v7.4.entrez.gmt” and “c5.go.v7.4.entrez.gmt” were extracted from MSigDB database (www.gesa-msigdb.org/gesa/msigdb/). GO terms and KEGG pathways with |NES| > 1, NOM p < 0.05, and q < 0.25 were considered to be markedly enriched.

### The relationship between immune infiltration and autophagy

To characterize the relationship between immune infiltration and autophagy in LUAD, we first performed ssGSEA to analyze the infiltration of 24 immune cells in the low- and high-risk groups. The Wilcoxon test was used to identify differentially infiltrated immune cells between the low- and high-risk groups with a p-value < 0.05. Furthermore, the correlations between the expressions of prognostic ARGs and the infiltration levels of 24 immune cells were calculated.

### Cell culture and irradiation treatment

Human lung adenocarcinoma cell lines A549 and PC9 were obtained from the Institute of Biochemistry and Cell Biology (Shanghai, PR China). Cells were placed in T-75 flasks and cultured in RPMI-1640 basal media (BASALMEDIA, PR China) with 10% fetal bovine serum (CELLIGENT, New Zealand) in an incubator with 5% CO2 and at 37°C. Cells were irradiated with RS2000 Biological X-ray Biological Irradiator (Georgia, USA) with an energy of 280 keV at a dose rate of 1.8 Gy/min at room temperature in the Radiation Therapy Center of the Third Affiliated Hospital of Kunming Medical University. To establish radiotherapy-resistant cell lines, A549 and PC9 cells were exposed to a repeated fraction of 4 Gy X-irradiation at a total dose of 56Gy. Two subconfluent 75-cm^2^ flasks of cells were exposed to repeated fractions of 4 Gy X-irradiation. The total number of cells present in the flasks at the time of the first irradiation was approximately 1 × 10^7^. A time interval of between 3 and 5 days was allowed to expose irradiated cells that had grown to 80% density, so that the cells could survive. In this way, the cell number present at each irradiation was kept roughly constant. Fresh medium was added with each irradiation, and after every five fractions, the contents of the two flasks were pooled, and a small fraction was plated out separately. These cells were used for clonogenic assays and to establish frozen stocks. The cell lines that acquired resistance to radiotherapy by continuous low-dose irradiation were named A549IR and PC9IR, respectively.

### Quantitative real-time PCR (qRT-PCR)

Total RNA was extracted from the cell lines in each flask *via* TRIzol, and reversed transcription into cDNA was performed using the SYBR Green Master Mix kit. The β-Actin gene was used as an endogenous control. The primer sequences were as follows: *AURKA*, Forward 5-GGTCAGTACATGCTCCATCTT CCAG-3’, Reverse 5’ - AGAACTCCAAGGCTCCAGAGATCC-3’; *NAPSA*, Forward 5’ -CTTCAGTGTGCCCTGCTGGTTAC-3’, Reverse 5’ - CATCTACCCGCCCAGTTCCATATTG-3’; and *SHC1*, Forward 5’ -TGAGGGTGTGGTTCGGACTAAGG-3’, Reverse 5’ -CCGCAGA GATGATGGGCAAGTG-3’. Data were analyzed using the comparative Ct method (2^-△△Ct^).

### Western blot assay

The cell culture flasks were washed with ice-cold PBS, then ice-cold lysis buffer (Solarbio, China) was added. Cell lysate was collected at 12,500 rpm and 4°C, and boiled for 10 min in loading buffer. Proteins were resolved on 12.5% SDS-PAGE gels, transferred onto PVDF (0.2 µm) membranes, and blocked with 5% nonfat dry milk. The following primary antibodies were used: 1:1000 for LC3B (#43566, CST), 1:1000 for SQSTM1/p62 (#8025, CST), and 1:5000 for GAPDH (#5174, CST). Primary antibodies to LC3b and p62 were diluted following the manufacturer’s instructions and incubated overnight at 4°C on a shaker at a slow speed. Then, secondary antibodies were incubated at room temperature for 2 h on a shaker at a slow speed. The PVDF membranes were washed three times with PBS, and the ECL Plus kit was used to visualize the immunoreactive process. Relative protein levels were normalized according to GAPDH concentration.

### Colony formation assay

300 cells were cultured in 60-mm dishes according to the pre-experiment, and after the cells fully adhered to the wall at 24 h after plate laying, the unadhered cells were removed by discarding the old medium and replacing it with fresh complete medium. Cells were allowed to continue to proliferate *in vitro* for more than six generations. After approximately 12–14 days, the cells were washed using PBS, fixed with 4% paraformaldehyde, and finally stained with crystal violet. The number of clones with > 50 cells was counted. Plating efficiency (PE)was measured for each cell line, and Surviving fractions were calculated using the equation SF = (number of colonies formed)/(number of cells seeded × plating efficiency for sham irradiated group)×100%.

### Statistical analysis

The data were displayed as the average ± standard deviation (SD) from the three independent trials, and statistical analysis was performed using SPSS 20.0 software. Graphics were created using GraphPad Prism 8 software. One-way analysis of variance analysis was carried out in multiple groups, and the matched Student’s Test was used in two groups, respectively.

## Results

### Identification of key genes involved in LUAD radiotherapy

**Figure 2:**
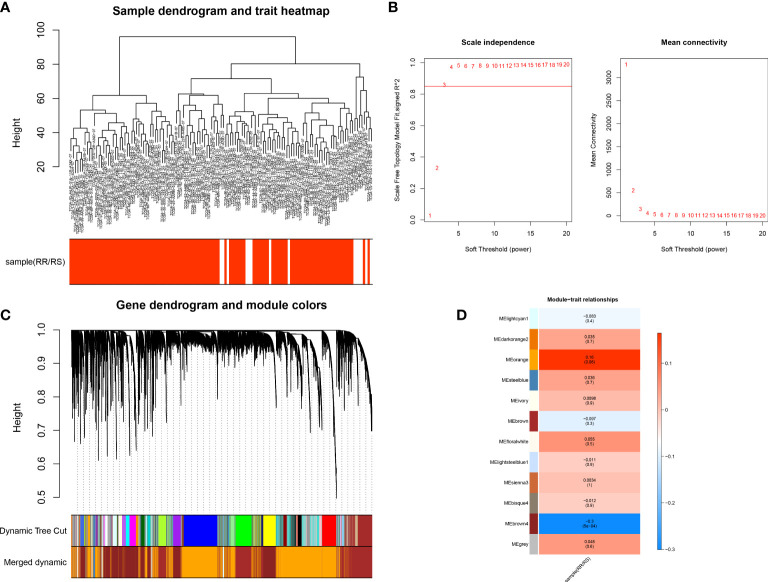
Identification of key genes in the radiotherapy of LUAD. **(A)** Sample Clustering Chart. **(B)** Selection of the optimal soft threshold power. **(C)** Module clustering dendrogram. **(D)** Heat map of the relationship between gene modules and traits, using the type of radiotherapy effect as the phenotype.

To identify the genes associated with radiotherapy, we performed WGCNA. The sample clustering result revealed no outlier samples, and the sample dendrogram and trait heatmap are shown in **Figure 2A**. The optimal soft threshold value was 3, in which R^2^ was approximately 0.85 (**Figure 2B**). We finally identified 11 modules after merging similar modules (**Figure 2C**), and the brown4 module was most relevant to radiotherapy (cor = -0.3, p-value < 0.01) (**Figure 2D**). Thus, 1,900 genes in the brown4 module were used for the downstream analysis.

### Identification of DERRAGs in LUAD

**Figure 3:**
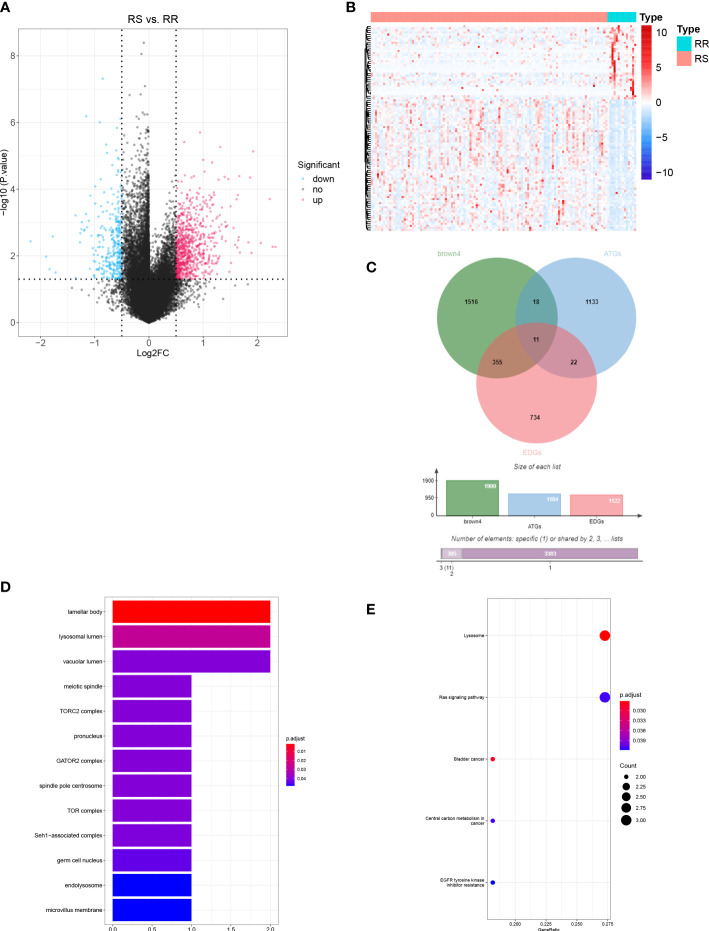
Identification of DERRAGs in LUAD. **(A)** Volcano map of RS vs. RR differentially expressed genes (DEGs) in LUAD. **(B)** Heat map of RS vs. RR DEGs in LUAD (Top 100). **(C)** The Venn diagram of DEGs, ATGs, and key module genes (brown4). **(D)** GO enrichment analysis of DERRAGs. **(E)** KEGG enrichment analysis of DERRAGs.

A total of 1121 DEGs were identified between the RS and RR groups (**Table S2** and **Figure 3A**). The expressions of the top 100 DEGs are displayed in the heatmap (**Figure 3B**). After intersecting 1121 DEGs with 1900 module genes and 1183 ATGs, *AURKA, CTSV, DAPK1, FGFR3, NAPSA, PLCH1, RGL1, SESN3, SHC1, SLC7A5*, and *SMPD1* were identified as DERRAGs in LUAD (**Figure 3C**). The DERRAGs were significantly enriched in 13 cellular components, including lamellar body, lysosomal lumen, vacuolar lumen, meiotic spindle, TORC2 complex, pronucleus, GATOR2 complex, spindle pole centrosome, TOR complex, Seh1-associated complex, germ cell nucleus, endolysosome and microvillus membrane (**Figure 3D**), and pathways of lysosome, Ras signaling, and bladder cancer (**Figure 3E**).

### Construction and validation of the autophagy-related risk score model

**Figure 4:**
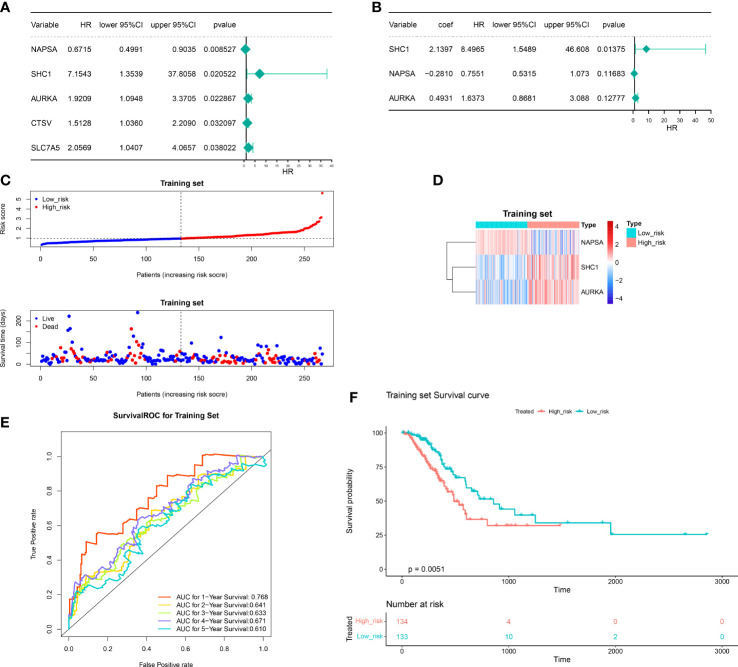
Construction of the autophagy-related risk score model. **(A)** Univariate forest plot of the correlation between the expression of DERRAGs and the overall survival of LUAD patients. **(B)** Multifactorial forest plot of the correlation between expression of DERRAGs and overall survival of LUAD patients. **(C)** The validation set high and low-risk group curves. **(D)** Heat map of model gene expression in the high and low-risk groups in the training set. **(E)** Training set survival ROC curve. **(F)** KM survival curves for the high and low-risk groups in the training set.

**Figure 5:**
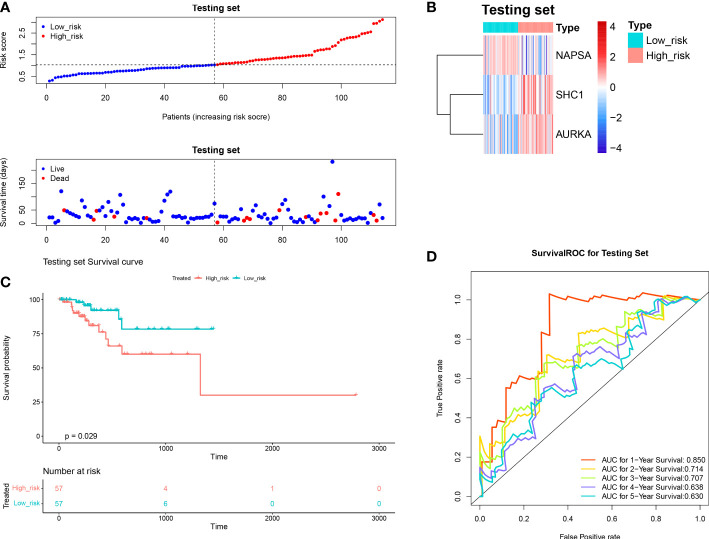
Validation of the autophagy-related risk score model (in the internal TCGA). **(A)** The test set high and low-risk group curves. **(B)** Heat map of model gene expression in the high and low-risk groups of the test set. **(C)** The test set high and low-risk group KM survival curves. **(D)** Test Set Survival ROC Curve.

**Figure 6:**
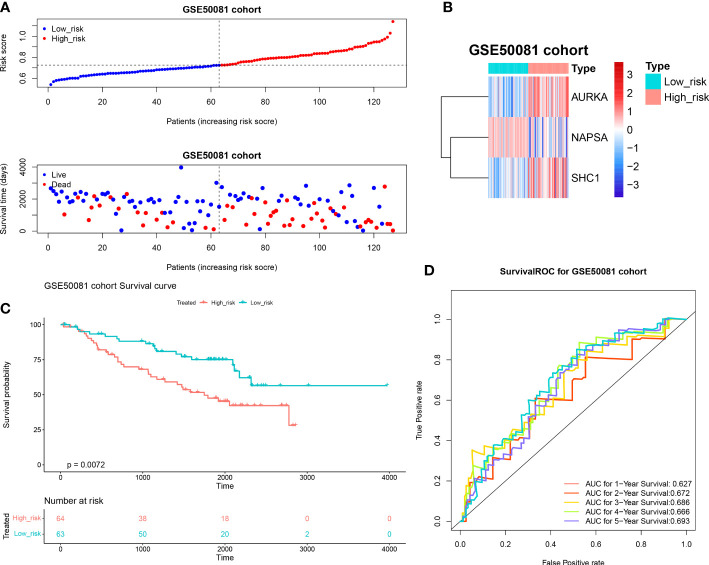
Validation of the autophagy-related risk score model (external GSE50081 dataset). **(A)** The validation set high and low-risk group curves. **(B)** Heat map of model gene expression in the high and low-risk groups in the validation set. **(C)** KM survival curves for the high and low-risk groups in the validation set. **(D)** Validation set survival ROC curve.

We investigated the prognostic value of DERRAGs. By univariate Cox regression analysis, *NAPSA, SHC1, AURKA, CTSV*, and *SLC7A5* were closely related with OS of LUAD (**Figure 4A**). Then, five genes were input into multivariate Cox regression, and *SHC1, NAPSA*, and *AURKA* were further selected to establish the autophagy-related risk model (**Figure 4B**). According to the coefficients and expressions of *SHC1, NAPSA*, and *AURKA*, the risk scores were calculated, and the cases were divided into two risk groups (**Figure 4C**). The expressions of *SHC1* and *AURKA* were significantly higher, while the expression of *NAPSA* was lower in the high-risk group (**Figure 4D**). Furthermore, a remarkable difference in survival was also identified between both risk groups (**Figure 4E**). The predictive efficiency of the risk model was investigated by ROC curves, and the risk model showed moderate accuracy with areas under the curves (AUC) > 0.6 (**Figure 4F**). The consensus results were also obtained in the internal TCGA (**Figure 5**) and external GSE50081 datasets (**Figure 6**).

### The autophagy-related nomogram was established in LUAD

**Figure 7:**
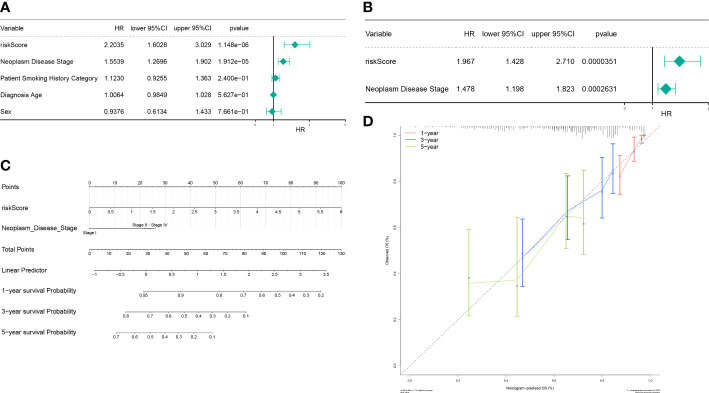
The autophagy-related nomogram was established in LUAD. **(A)** Independent prognosis-univariate Cox Forest plot. **(B)** Independent prognosis-multifactorial Cox Forest plot. **(C)** Risk model and disease staging constructs column line graphs. **(D)** 1-, 3-, and 5-year calibration curve.

Next, the independent prognostic factors were investigated by univariate analysis. We discovered that risk score, neoplasm disease stage, patient smoking history category, diagnosis age, and sex were significantly related to prognosis (**Figure 7A**). These factors were then subjected to multivariate analysis, and risk score and neoplasm disease stage were still significantly related with prognosis (**Figure 7B**), indicating that they were independent prognostic factors of LUAD. Furthermore, we used risk score and neoplasm disease stage to construct a nomogram to predict survival (**Figure 7C**). The calibration curves showed that the predicted 1- and 3- year OS were close to the actually observed OS (**Figure 7D**).

### The risk score had a close relationship with cell proliferation

**Figure 8:**
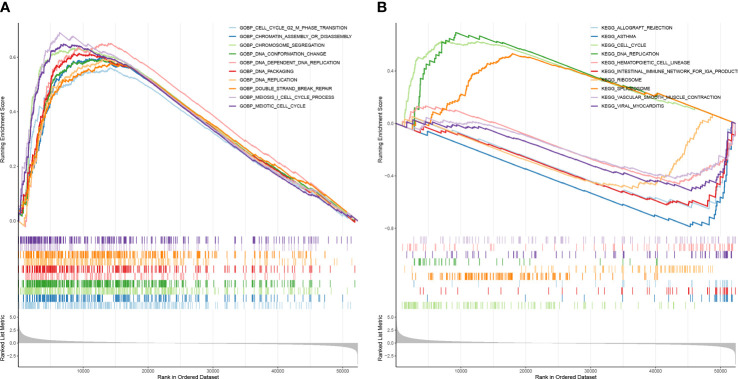
The risk score had a close relationship with cell proliferation. **(A)** GSEA enrichment analysis for high and low-risk groups—GO enrichment analysis. **(B)** CSEA enrichment analysis for high and low-risk groups–KEGG pathway analysis.

To elucidate the relevant pathways of these prognostic signatures in LUAD, the GSEA analysis was performed. The genes in the high-risk group were enriched in biological processes related to cell proliferation, including cell cycle G2/M phase transition, DNA conformation change, chromatin assembly or disassembly, DNA-dependent DNA replication, chromosome segregation, DNA packaging, DNA replication, meiosis I cell cycle process, double strand break repair, and meiotic cell cycle (**Figure 8A**). Consistent with the GO results, cell proliferation-related pathways were also enriched in the high-risk group when KEGG was applied (**Figure 8B**).

### The immune microenvironment was different between the low- and high-risk groups

**Figure 9:**
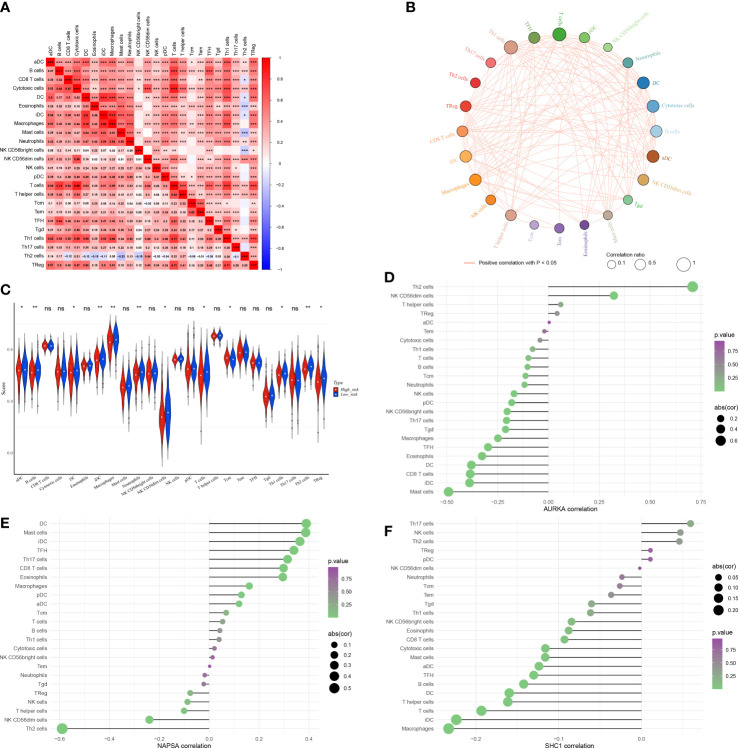
The immune microenvironment was different between the low and high-risk groups. **(A)** Heat map of immune cell correlation analysis in the high and low-risk groups. **(B)** Immunocellular correlation analysis Net plot of the high and low-risk groups. **(C)** Immune cell infiltration in the high and low-risk groups - Violin diagram. **(D–F)** *AURKA, NAPSA*, and *SHC1* prognostic biomarker genes, and immune cell correlation. *p < 0.05, **p < 0.01, and ***p < 0.001. "ns" indicates P value ≥ 0.05, not statistically significant.

Considering the importance of the immune microenvironment in LUAD progression, we examined the immune infiltration landscape of patients in both risk groups. We observed that there were moderate or strong correlations between CD8 T cells and cytotoxic cells, iDC and DC, macrophages and DC, macrophages and iDC, NK CD56dim cells and cytotoxic cells, T cells and B cells, T cells and cytotoxic cells, Th1 cells and cytotoxic cells, Th1 cells and DC, Th1 cells and macrophages, Th1 cells and T cells, Treg and T cells, and Treg and Th1 cells in LUAD (**Figures 9A, B**). Moreover, the infiltration of aDC, macrophages, B cells, NK CD56dim cells, DC, Tcm, iDC, Treg, T cells, neutrophils, Th1 cells, and Th2 cells were significantly different in both risk groups (**Figure 9C**). AURKA expression had a significantly positive correlation with Th2 cells abundance (**Figure 9D**), NAPSA expression had a significantly positive or negative correlation with Th2 cells and DC infiltration (**Figure 9E**). Although SHC1 expression had significantly positive or negative correlations with multiple differentially infiltrated immune cells, their correlations were very weak (|cor| < 0.3, **Figure 9F**).

### Validation of the relationship between SHC1, NAPSA, and AURKA genes, radiotherapy sensitivity, and autophagy by constructing resistant cell lines compared with primary cells

**Figure 10:**
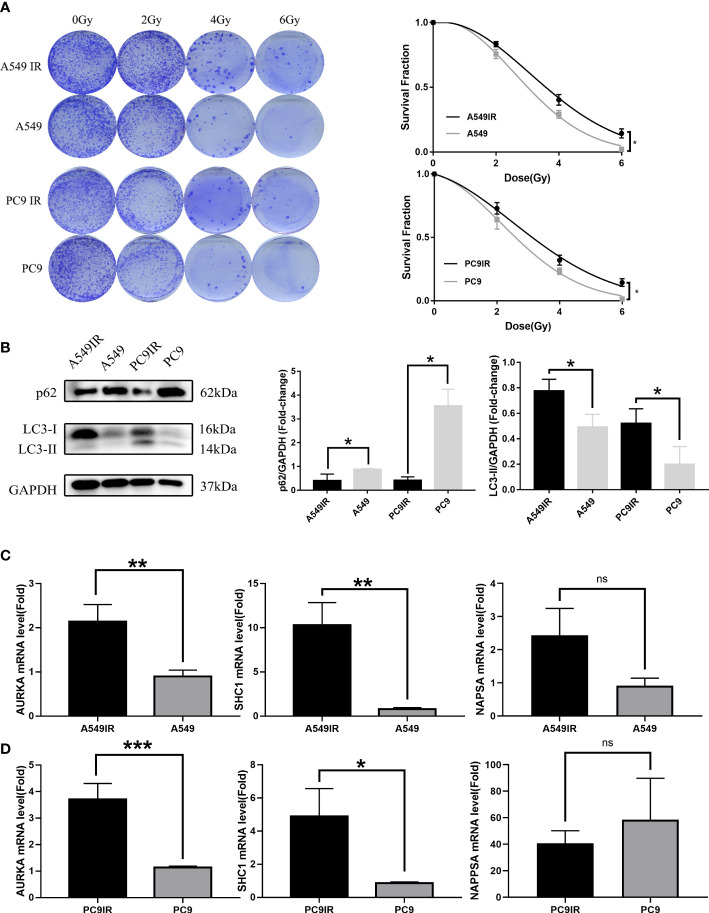
Validation of the relationship between *AURKA, SHC1*, and *NAPSA* genes and radiotherapy sensitivity, and autophagy by constructing resistant cell lines compared with primary cells. **(A)** Colony formation assay reveal constructed resistance cells with enhanced radioresistance. **(B)** The western blotting assay showed increased levels of resistant cell autophagy. **(C, D)** Quantitative Real-time PCR verified significant differences in radiotherapy autophagy-related genes (*AURKA, SHC1*) between radiotherapy-resistant and primary cells. *p < 0.05, **p < 0.01, and ***p < 0.001. "ns" indicates P value ≥ 0.05, not statistically significant.

Radioresistant cells were constructed by interval irradiation of cells. In this study, we found that the radiation-resistant cell lines were resistant to radiation in clone formation experiments, and their cell survival rate after IR was obviously above the survival rate of primary cells. (**Figure 10A**).

Elevated levels of autophagy in radioresistant cells were found by western blot assay (**Figure 10B**). The expression of RRAGs (SHC1, AURKA) was higher in radioresistant cells than in primary cells as verified by qRT-PCR (**Figures 10C, D**). However, no significant difference in *NAPSA* gene expression was observed between radioresistant cells and primary cells. The *NAPSA* gene is highly expressed mainly in LUAD and kidney cancer; therefore, the *NAPSA* gene may be closely related to tissue typing. The primary cells that we used to construct radioresistant cells were all LUAD cells; therefore, there was no significant difference in *NAPSA* gene expression between the radioresistant and primary cells.

## Discussion

In recent years, many large-scale clinical studies involving patients with LUAD have confirmed improvements in survival time and quality of life associated with radiotherapy, and these results have helped to set radiotherapy as the standard of care for LUAD (8, 9). Radiotherapy not only reduces tumor burden but also triggers anti-tumor immunity and reprograms the tumor microenvironment (10, 11). However, radiation resistance remains a critical limiting factor in the efficacy of LUAD therapy. Previous research has shown that targeting autophagy-related genes can improve tumor radiotherapy efficacy while reducing cancer toxicity (12). Autophagy and radiotherapy resistance are thus closely linked. In this study, we obtained three autophagy-related genes that affect LUAD radiotherapy and prognosis. The survival of LUAD patients was predicted by constructing a risk model and nomogram.

*SHC1* can encode three major isoforms (p46SHC, p52SHC, and p66SHC), all of which have highly conserved structural domains unique to the Shc family (13, 14). One of the functions of SHC1 proteins, notably p66SHC, is to modulate redox signaling, and it has long been known to have a role in oxidative stress-induced apoptosis and mammalian lifespan. In A549 cells under nutrient-restricted settings, p66SHC might be significant in coordinating the regulation of the autophagic process with the anti-apoptotic process, leading to therapeutic resistance (15). Moreover, p66SHC can affect energy metabolism by disrupting mitochondrial function and reducing ATP production, which subsequently activates the AMPK/mTOR signaling pathway-mediated increase in autophagic flux (16). *In vivo*, UV irradiation can induce oxidative stress in mice by activating the JNKs signaling pathway through phosphorylation of the p66SHC serine 36 site, and mice lacking p66SHC are thus less susceptible to this effect, resulting in a longer lifespan (17). An increased expression of *SHC1* strongly relates to worse outcomes and treatment resistance in LUAD. According to studies, *SHC1* expression was upregulated in LUAD tissues, correlated with poorer OS, and associated with LUAD stages (18). Therefore, p66SHC may increase resistance to radiotherapy and other treatments by inducing ROS and autophagy, thereby affecting therapeutic efficacy. Similar results were obtained in our study with enhanced autophagy and *SHC1* expression in radiotherapy-resistant cell lines (A549IR and PC9IR). Therefore, radiotherapy-resistant cell lines may overexpress *SHC1* to mediate elevated levels of autophagy and thus contribute to the survival of tumor cells after irradiation by affecting radiotherapy sensitivity, resulting in radiotherapy resistance. Thus, targeting *SHC1* may be a novel approach to improve the efficacy of LUAD radiation therapy.

AURKA belongs to the serine/threonine kinase family, and its activation is necessary to modulate cell mitosis (19). A growing number of studies suggest that when AURKA is aberrantly expressed, it may be an oncogene involved in tumorigenesis. Overexpression of AURKA has been detected in many tumor cells and tissues, including LUAD (20). Moreover, AURKA expression varies among NSCLC subtypes, and it was found to be primarily upregulated in moderately and poorly differentiated lung tumors (21). AURKA is significantly upregulated in a wide range of tumor tissues, and increased expression in LUAD is associated with worse treatment outcomes, with individuals with this characteristic having a lower OS rate (22). In the treatment of LUAD, there is increasing evidence that AURKA inhibitors can improve the response to radiation therapy (23–25). Activation of the AURKA-PLK1 signaling pathway can bypass the ATM-dependent G2 checkpoint and cause impaired cellular damage repair and genomic instability, thereby triggering tumorigenesis (26). In contrast, interference with the AURKA-CXCL5 signaling pathway can mediate autophagic cell death in LUAD, thereby enhancing radiosensitivity (27). Our study showed that A549IR and PC9IR showed increased autophagy flux and AURKA expression. Therefore, we speculate that high expression of AURKA may increase radiotherapy resistance by increasing autophagy.

Napsin A is encoded by the *NAPSA* gene, which is a sensitive and specific marker for LUAD (28, 29). In different studies, the positive rate of Napsin A in LUAD ranged from 58% to 91% in different studies (30, 31). In lung cancer, Napsin A was specifically and highly expressed in LUAD, but not in squamous lung cancer (32), small cell lung cancer (33), or carcinoid tumors (34). It was found that NAPSA was negatively correlated with the degree of transformation in a tumor (31). A recent study showed that overexpression of NAPSA inhibits integrin signaling to reverse EMT, thus reversing the susceptibility of gefitinib-resistant A549 cell lines to regain drug treatment (35). Further studies found that LUAD patients who had elevated Napsin A expression had a better survival rate (36). In addition, Napsin A deficiency is also a risk indicator for worse postoperative prognosis in LUAD patients (37). Therefore, NAPSA may be an effective therapeutic target for lung adenocarcinoma. To our knowledge, the sensitivity of NAPSA to radiotherapy for lung adenocarcinoma has not been reported. Our study found that NAPSA expression decreased obviously in the radiotherapy-resistant group of patients. Combining previous studies with our results, we hypothesize that NAPSA may influence the effect of radiotherapy in LUAD patients through the EMT-regulated cell proliferation process.

To resolve the molecular mechanism of the three genes regulating LUAD, we performed GSEA analysis and found that the risk score was closely related to cell proliferation. The GO and KEGG enrichment of DERRAGs is mainly related to lysosomes, cell cycle transition, DNA replication, double-strand break repair, and Ras signaling pathway. Autophagy is a multi-phased dynamic process regulated by various ARGs, and recent studies have found that autophagy may influence the effectiveness of radiation therapy (38). Cellular damage caused by radiation includes DNA single-strand breaks and double-strand breaks that activate DNA damage response (DDR) and thus affect the level of autophagic fluxes (39). The DDR pathway activates cell cycle checkpoints causing cell cycle arrest, thus facilitating damage repair. Meaningfully, chaperone-mediated autophagy has been reported to regulate Chk1 degradation during DNA damage in response to DDR (40). The cell cycle checkpoint is a key factor that can influence radiosensitivity by affecting cell cycle arrest and damage repair. Like cell cycle checkpoints, autophagy can be considered a highly conserved cytoprotective mechanism in most cases. A new study found that resistance to radiotherapy due to radiation-induced autophagy in human pancreatic cancer cell lines is considerably dependent on G2 checkpoint activation (41). Thus, there is a crosstalk between G2 checkpoint activation and radiation-induced autophagic processes. Therefore, when DNA is damaged and repaired after radiotherapy, the autophagy and cell cycle checkpoint pathways may regulate the cell proliferation process by cross-talking between them.

With the advancement in the understanding of radiobiology, the pursuit of radiotherapy efficacy has changed from “radiation damage” to “radiation effect” and from simply causing tumor damage to improving the immune microenvironment after radiation. Radiation therapy can reshape the tumor’s immunological microenvironment and change the proportion of immune cells infiltration. Moreover, cumulative evidence indicates that autophagic activity can regulate immune cell infiltration through the regulation of the innate and adaptive immune systems. Therefore, it is possible to use autophagy as a new immunomodulatory strategy to improve the efficacy of radiotherapy in LUAD patients. We examined the immune infiltration of the patients and found that aDC, B cells, DC, iDC, macrophages, neutrophils, NK CD56dim cells, T cells, Tcm, Th1 cells, Th2 cells, and Treg infiltration were markedly different between the RS and RR groups. DCs are the most functional antigen-presenting cells (APC), mature DCs can effectively activate the initial T cells and are at the center of initiating, regulating, and maintaining the immune response (40). DC cell subsets induce different specific T cell responses and thus determine whether they cause immune activation or immunosuppression (42). Several studies have suggested that the mechanisms of immunosuppression may be caused by dysregulation of the Th1/Th2 balance. Although Th1 cells generate cytokines that act as a suppressor against a microenvironment that promotes tumor growth (43), conversely Th2 cells release cytokines that cause immunosuppression, which can lead to the immune escape of tumor cells (44). Experimental animal studies have found that IR triggers a sustained Th2 immune response, thereby altering the Th1/Th2 balance, resulting in immune-suppression (45). Our study also found that *SHC1* and *AURKA* expression was elevated in the RR group, positively correlated with Th2, and negatively correlated with Th1, while *NAPSA* expression was elevated in the RS group, positively correlated with Th1, and negatively correlated with Th2. Therefore, we presume that these three prognostic signature genes are associated with the immunological modulation of the Th1/Th2 shift after radiotherapy.

With the advent of immunotherapy, it is becoming increasingly important to study how autophagy changes the tumor immune microenvironment, thereby influencing the therapeutic outcome of cancer. Both Th1 and Th2 cells are induced by autophagy, but autophagy induces more effector Th2 cells than Th1 cells, thereby reducing the ratio of Th1/Th2 cells and inducing suppression of the tumor immune microenvironment (46). Recent evidence indicates that radiation-induced immunosuppression may be caused by a reduction in tumor-infiltrating CD8+ DCs and a decrease in the Th1/Th2 ratio (47). Therefore, IR-induced autophagy may cause a Th1/Th2 imbalance and thus create an immunosuppressive environment. Macrophages are recruited at the site of irradiation after IR (48). Recent studies have shown that high-dose radiation promotes anti-inflammatory activation *via* macrophages (49), and low-dose radiation with immunotherapy induces pro-inflammatory activation of macrophages (50). Therefore, IR can activate macrophages and thus affect the efficacy of radiotherapy. Our study observed that macrophages were significantly different in the two risk groups. However, T cells may also suppress the tumor microenvironment in LUAD. A subpopulation of CD4+ T cells, the regulatory T cells (Tregs), is a key player in suppressing anti-tumor immunity (51). Radiotherapy can increase Tregs cell infiltration, thereby triggering immunosuppression and contributing to a pro-tumorigenic phenotype (52). One of the current strategies for enhancing radiation-mediated anti-tumor immunity is Treg depletion (53). However, recent studies have found that deletion of two important genes in autophagy, *Atg7* or *Atg5*, leads to loss of Treg cells in an inflammation-activated environment. As a result, autophagy interacts with environmental signaling and metabolic homeostasis to protect Treg cells in an inflammation-activated environment (54). Thus, autophagy may affect the antitumor effect by increasing Treg cell infiltration after radiotherapy.

In conclusion, this study identified for the first time three autophagy genes associated with LUAD radiotherapy and prognosis, discussed the underlying molecular mechanisms, and developed a predictive model that can effectively predict the prognosis of LUAD. *SHC1, NAPSA*, and *AURKA*, which are strongly linked to immune cell infiltration and are relevant biomarkers that may have great predictive power, are potentially significant for the efficacy and prognostic improvement of radiotherapy in patients with LUAD. Furthermore, we confirmed that radiotherapy resistance in LUAD is partially due to elevated levels of autophagy by comparing control and IR-resistant cells. However, the sample data of this study were obtained from the TCGA and GEO databases, and different radiation doses and radiotherapy fractionation schedules mediating autophagy and remodeling of the immune microenvironment may have different effects. Currently, no optimal radiation doses and segmentation scheme have been identified for combining immunotherapy. Next, we will investigate the relationship between autophagy and immunotherapy in LUAD with different radiation doses and fractionation schemes. Furthermore, since there are very few LUAD patients treated with postoperative radiotherapy, tissue samples are difficult to collect; thus, this study can only be verified by constructing a radiotherapy-resistant cell line.

## Data availability statement

The original contributions presented in the study are included in the article/**Supplementary Material**. Further inquiries can be directed to the corresponding authors.

## Author contributions

Conception, design: JG, FL, and WL. Acquisition and interpretation of data: JG, JY, RW, and LL. Analyzation of data: JG, JY, YX, and LC. Visualization of results: LL and LW. Initial manuscript writing: JG, FL, and JY. Revision of manuscript: WL and LC. All authors read and approve the final manuscript.

## Funding

This study was funded by the National Natural Science Foundation of China (Nos. 81860536 and 82060558), Yunnan Fundamental Research Projects (Nos. 202001AS70011, 202101AY070001-162 and 202201AY070001-221), Ten-thousand Talents Program of Yunnan Province (Yunling scholar, Youth talent), Yunnan Provincial Training Funds for Middle-Young Academic and Technical Leader candidate (No. 202005AC160025), Yunnan Provincial Training Special Funds for High-level Health Technical Personnel (Nos. L-2018001 and D-2019030), Yunnan Provincial Education Department Scientific Research Fund (Nos. 2022Y220).

## Conflict of interest

The authors declare that the research was conducted in the absence of any commercial or financial relationships that could be construed as a potential conflict of interest.

## Publisher’s note

All claims expressed in this article are solely those of the authors and do not necessarily represent those of their affiliated organizations, or those of the publisher, the editors and the reviewers. Any product that may be evaluated in this article, or claim that may be made by its manufacturer, is not guaranteed or endorsed by the publisher.
