# Supplementary material for: The role of radiotherapy-related autophagy genes in the prognosis and immune infiltration in lung adenocarcinoma
Source: Front Immunol. 2022 Oct 13;13:992626. doi: 10.3389/fimmu.2022.992626 (PMC9606704; doi:10.3389/fimmu.2022.992626)
Supplement: Supplementary file 2 [file Table_2.docx]

| **Symbol** | **logFC** | **AveExpr** | **t** | **P.Value** | **adj.P.Val** | **B** |
| --- | --- | --- | --- | --- | --- | --- |
| MARCHF11 | -0.8500 | 0.1258 | -5.8042 | 4.84E-08 | 8.92E-04 | 7.6536 |
| RAB26 | -1.1567 | 1.4373 | -5.2397 | 6.46E-07 | 3.32E-03 | 5.1322 |
| RIPPLY2 | -0.5217 | 0.1234 | -5.1968 | 7.82E-07 | 3.33E-03 | 4.9470 |
| ALDOA | -0.9059 | 7.7401 | -5.1447 | 9.85E-07 | 3.89E-03 | 4.7239 |
| QSOX2 | -0.5897 | 2.8513 | -5.0559 | 1.45E-06 | 4.35E-03 | 4.3465 |
| FCGRT | 0.9420 | 5.4780 | 4.9844 | 1.98E-06 | 4.35E-03 | 4.0461 |
| S1PR2 | 0.6512 | 2.7552 | 4.8300 | 3.84E-06 | 6.44E-03 | 3.4068 |
| UBE2QL1 | -0.7898 | 0.4465 | -4.7887 | 4.58E-06 | 6.80E-03 | 3.2380 |
| TNFSF10 | 1.3146 | 4.9073 | 4.7504 | 5.38E-06 | 7.43E-03 | 3.0826 |
| RP11-114B7.6 | -0.6025 | 0.2655 | -4.6926 | 6.85E-06 | 8.74E-03 | 2.8496 |
| NDNF | 1.9226 | 3.7023 | 4.6754 | 7.36E-06 | 8.84E-03 | 2.7808 |
| ADRA2C | -0.7718 | 0.7639 | -4.6486 | 8.22E-06 | 9.28E-03 | 2.6738 |
| ASH1L-AS1 | -0.5701 | 1.5604 | -4.5666 | 1.15E-05 | 1.14E-02 | 2.3490 |
| AACSP1 | -0.5592 | 0.1936 | -4.5442 | 1.26E-05 | 1.16E-02 | 2.2607 |
| TRAM1 | 1.0005 | 6.6491 | 4.5332 | 1.32E-05 | 1.16E-02 | 2.2177 |
| MOXD1 | 1.1661 | 2.7929 | 4.4900 | 1.58E-05 | 1.24E-02 | 2.0490 |
| GALNT13 | -0.6727 | 0.4388 | -4.4453 | 1.89E-05 | 1.40E-02 | 1.8760 |
| ZNF768 | -0.5667 | 4.4606 | -4.3587 | 2.67E-05 | 1.80E-02 | 1.5440 |
| GJA5 | 1.0254 | 2.9538 | 4.3314 | 2.97E-05 | 1.87E-02 | 1.4404 |
| SUV39H2-DT | -0.5679 | 0.9320 | -4.3293 | 3.00E-05 | 1.87E-02 | 1.4323 |
| SNORD88A | -0.5165 | 0.4929 | -4.3282 | 3.01E-05 | 1.87E-02 | 1.4281 |
| TEX101 | -0.5923 | 0.1826 | -4.2742 | 3.72E-05 | 2.03E-02 | 1.2246 |
| TNFSF12 | 0.6637 | 3.9269 | 4.2659 | 3.85E-05 | 2.03E-02 | 1.1938 |
| ATP13A4 | 1.6670 | 2.5836 | 4.2522 | 4.06E-05 | 2.03E-02 | 1.1423 |
| HNMT | 0.8280 | 3.3326 | 4.2512 | 4.08E-05 | 2.03E-02 | 1.1386 |
| KCNK5 | 1.3966 | 4.0136 | 4.2392 | 4.27E-05 | 2.03E-02 | 1.0938 |
| SLFN5 | 0.7864 | 3.4583 | 4.2271 | 4.48E-05 | 2.03E-02 | 1.0487 |
| DPP4 | 1.8667 | 4.1332 | 4.2259 | 4.50E-05 | 2.03E-02 | 1.0443 |
| TMPRSS2 | 1.4656 | 4.1044 | 4.1996 | 4.98E-05 | 2.12E-02 | 0.9467 |
| MIR210 | -0.8894 | 0.9118 | -4.1967 | 5.04E-05 | 2.13E-02 | 0.9362 |
| CPVL | 1.2843 | 3.1263 | 4.1885 | 5.20E-05 | 2.15E-02 | 0.9058 |
| CSGALNACT2 | 0.5389 | 3.2348 | 4.1590 | 5.83E-05 | 2.18E-02 | 0.7972 |
| TMEM50B | 0.6049 | 3.9176 | 4.1577 | 5.86E-05 | 2.18E-02 | 0.7923 |
| OR51E1 | -0.5170 | 0.2474 | -4.1019 | 7.25E-05 | 2.41E-02 | 0.5885 |
| LINC01139 | -0.9902 | 0.6839 | -4.0680 | 8.25E-05 | 2.50E-02 | 0.4657 |
| RP11-432J22.2 | -0.5274 | 1.7224 | -4.0636 | 8.39E-05 | 2.50E-02 | 0.4497 |
| DACT3 | 0.5204 | 1.0559 | 4.0377 | 9.26E-05 | 2.62E-02 | 0.3563 |
| CLEC7A | 0.9355 | 2.6493 | 4.0342 | 9.38E-05 | 2.64E-02 | 0.3440 |
| H2AC11 | -0.6923 | 0.6469 | -4.0281 | 9.60E-05 | 2.67E-02 | 0.3222 |
| UNKL | -0.5092 | 1.8925 | -4.0165 | 1.00E-04 | 2.73E-02 | 0.2807 |
| CD59 | 0.7558 | 5.9457 | 3.9990 | 1.07E-04 | 2.83E-02 | 0.2179 |
| SLC25A25-AS1 | -0.5649 | 1.5076 | -3.9697 | 1.20E-04 | 2.98E-02 | 0.1137 |
| PTPRN | -0.8253 | 0.3026 | -3.9619 | 1.23E-04 | 3.04E-02 | 0.0863 |
| DRAM1 | 1.1971 | 4.7919 | 3.9570 | 1.25E-04 | 3.06E-02 | 0.0688 |
| SLC12A2 | 0.8856 | 2.6018 | 3.9520 | 1.28E-04 | 3.08E-02 | 0.0513 |
| IFIT5 | 0.6045 | 2.9522 | 3.9484 | 1.29E-04 | 3.08E-02 | 0.0387 |
| SCPEP1 | 0.9165 | 5.0446 | 3.9350 | 1.36E-04 | 3.09E-02 | -0.0088 |
| DARS2 | -0.6510 | 3.2290 | -3.9227 | 1.42E-04 | 3.09E-02 | -0.0520 |
| EMILIN2 | 0.7063 | 2.3450 | 3.9162 | 1.46E-04 | 3.09E-02 | -0.0747 |
| ZNF704 | 0.7075 | 1.7345 | 3.9147 | 1.47E-04 | 3.09E-02 | -0.0800 |
| CDC42EP1 | 1.2042 | 5.6059 | 3.9100 | 1.49E-04 | 3.11E-02 | -0.0964 |
| MFAP4 | 1.6393 | 4.9147 | 3.9095 | 1.49E-04 | 3.11E-02 | -0.0983 |
| SORD2P | -0.5095 | 0.6127 | -3.9060 | 1.51E-04 | 3.12E-02 | -0.1106 |
| ALOX5AP | 1.1156 | 4.1506 | 3.9033 | 1.53E-04 | 3.14E-02 | -0.1201 |
| ENTPD8 | -1.0807 | 1.3217 | -3.8972 | 1.56E-04 | 3.15E-02 | -0.1413 |
| FOXN4 | -0.5003 | 0.1521 | -3.8884 | 1.62E-04 | 3.21E-02 | -0.1721 |
| PSMB5 | -0.5782 | 6.0063 | -3.8723 | 1.71E-04 | 3.21E-02 | -0.2279 |
| AHR | 0.8816 | 4.7440 | 3.8720 | 1.72E-04 | 3.21E-02 | -0.2291 |
| KCTD12 | 1.0003 | 3.9345 | 3.8708 | 1.72E-04 | 3.21E-02 | -0.2333 |
| DCN | 1.1544 | 4.4110 | 3.8674 | 1.74E-04 | 3.21E-02 | -0.2450 |
| ASAP3 | 0.8087 | 2.9066 | 3.8597 | 1.79E-04 | 3.26E-02 | -0.2719 |
| SMURF2 | 0.7066 | 3.3010 | 3.8581 | 1.81E-04 | 3.26E-02 | -0.2774 |
| LUM | 1.1522 | 6.9640 | 3.8570 | 1.81E-04 | 3.26E-02 | -0.2810 |
| DDX3ILA1 | -0.5015 | 0.2711 | -3.8429 | 1.91E-04 | 3.37E-02 | -0.3298 |
| MEOX2 | 0.6937 | 1.2603 | 3.8385 | 1.94E-04 | 3.39E-02 | -0.3452 |
| RP11-588K22.2 | 0.7674 | 2.6535 | 3.8377 | 1.94E-04 | 3.39E-02 | -0.3479 |
| NAPSA | 2.2284 | 8.0879 | 3.8347 | 1.97E-04 | 3.40E-02 | -0.3583 |
| SAMHD1 | 0.8818 | 4.5066 | 3.8339 | 1.97E-04 | 3.40E-02 | -0.3611 |
| SFTA2 | 1.6146 | 7.2967 | 3.8298 | 2.00E-04 | 3.44E-02 | -0.3753 |
| TLR2 | 1.1274 | 3.4327 | 3.8161 | 2.10E-04 | 3.55E-02 | -0.4223 |
| MNDA | 1.0496 | 2.9598 | 3.8073 | 2.17E-04 | 3.65E-02 | -0.4524 |
| NR3C2 | 0.7576 | 1.7275 | 3.8057 | 2.19E-04 | 3.65E-02 | -0.4580 |
| APOBR | 0.7509 | 2.6660 | 3.7925 | 2.29E-04 | 3.73E-02 | -0.5030 |
| CHRNB2 | -0.5586 | 0.1567 | -3.7903 | 2.31E-04 | 3.73E-02 | -0.5107 |
| DOK4 | 1.0061 | 3.7292 | 3.7848 | 2.36E-04 | 3.76E-02 | -0.5293 |
| H2AC17 | -0.7596 | 0.8184 | -3.7729 | 2.46E-04 | 3.84E-02 | -0.5701 |
| CLEC5A | 0.7926 | 1.5859 | 3.7664 | 2.52E-04 | 3.91E-02 | -0.5922 |
| SLC5A3 | 0.6422 | 2.2785 | 3.7597 | 2.58E-04 | 3.92E-02 | -0.6150 |
| SLC40A1 | 1.0341 | 4.9157 | 3.7541 | 2.63E-04 | 3.92E-02 | -0.6338 |
| CHADL | 0.5443 | 1.4038 | 3.7517 | 2.65E-04 | 3.93E-02 | -0.6419 |
| LINC01876 | -0.5761 | 0.6664 | -3.7513 | 2.66E-04 | 3.93E-02 | -0.6435 |
| UBE2T | -0.9894 | 3.8184 | -3.7494 | 2.68E-04 | 3.95E-02 | -0.6498 |
| SMPD1 | 0.5755 | 4.0712 | 3.7426 | 2.74E-04 | 4.00E-02 | -0.6727 |
| CALCB | -0.6724 | 0.2135 | -3.7405 | 2.76E-04 | 4.00E-02 | -0.6800 |
| RARRES2 | 1.0719 | 5.0687 | 3.7353 | 2.82E-04 | 4.05E-02 | -0.6977 |
| OLR1 | 1.1867 | 3.2477 | 3.7310 | 2.86E-04 | 4.10E-02 | -0.7121 |
| LY75 | 0.5736 | 1.3441 | 3.7263 | 2.91E-04 | 4.10E-02 | -0.7279 |
| EHD2 | 0.9466 | 5.2214 | 3.7257 | 2.91E-04 | 4.10E-02 | -0.7300 |
| PRELP | 1.1675 | 3.5325 | 3.7238 | 2.93E-04 | 4.11E-02 | -0.7364 |
| MYLK | 0.7722 | 2.1622 | 3.7237 | 2.94E-04 | 4.11E-02 | -0.7368 |
| ISM1 | 0.8885 | 1.8978 | 3.7003 | 3.19E-04 | 4.30E-02 | -0.8152 |
| ZCCHC24 | 0.6198 | 2.7157 | 3.6978 | 3.22E-04 | 4.31E-02 | -0.8235 |
| SYT1 | -0.8828 | 0.9508 | -3.6958 | 3.24E-04 | 4.32E-02 | -0.8305 |
| RRAD | 1.5052 | 3.5980 | 3.6938 | 3.26E-04 | 4.34E-02 | -0.8371 |
| ANOS1 | 1.0764 | 2.1387 | 3.6879 | 3.33E-04 | 4.37E-02 | -0.8569 |
| COL8A2 | 0.9894 | 2.6080 | 3.6825 | 3.40E-04 | 4.37E-02 | -0.8750 |
| CACNB2 | -0.5077 | 0.5357 | -3.6725 | 3.52E-04 | 4.43E-02 | -0.9083 |
| PAPLN | 0.8363 | 2.3870 | 3.6719 | 3.53E-04 | 4.43E-02 | -0.9102 |
| LAMP3 | 1.3471 | 4.3090 | 3.6707 | 3.54E-04 | 4.43E-02 | -0.9141 |
| PLXDC2 | 0.7359 | 2.6482 | 3.6706 | 3.54E-04 | 4.43E-02 | -0.9146 |
| AC093323.3 | -0.5605 | 3.2286 | -3.6658 | 3.60E-04 | 4.45E-02 | -0.9304 |
| CCR1 | 0.8243 | 2.6443 | 3.6641 | 3.63E-04 | 4.45E-02 | -0.9362 |
| ADAP2 | 0.5904 | 2.4763 | 3.6590 | 3.69E-04 | 4.45E-02 | -0.9533 |
| AOC3 | 1.1241 | 3.3772 | 3.6583 | 3.70E-04 | 4.45E-02 | -0.9556 |
| FUCA1 | 0.7006 | 5.7576 | 3.6582 | 3.70E-04 | 4.45E-02 | -0.9558 |
| RP11-848P1.3 | 0.6186 | 1.9394 | 3.6452 | 3.88E-04 | 4.57E-02 | -0.9990 |
| GUCY1A1 | 0.7449 | 2.4114 | 3.6431 | 3.91E-04 | 4.59E-02 | -1.0059 |
| KCNH6 | -0.6246 | 0.1934 | -3.6427 | 3.91E-04 | 4.59E-02 | -1.0070 |
| TMEM25 | 0.5682 | 2.4856 | 3.6400 | 3.95E-04 | 4.60E-02 | -1.0162 |
| MGLL | 0.9818 | 4.3017 | 3.6388 | 3.96E-04 | 4.61E-02 | -1.0200 |
| GUCY1B1 | 0.6641 | 2.4335 | 3.6332 | 4.04E-04 | 4.64E-02 | -1.0386 |
| CLIC2 | 0.9764 | 3.4687 | 3.6327 | 4.05E-04 | 4.64E-02 | -1.0402 |
| BRI3BP | -0.5635 | 2.3941 | -3.6310 | 4.08E-04 | 4.64E-02 | -1.0459 |
| HCK | 0.9445 | 3.3268 | 3.6276 | 4.12E-04 | 4.67E-02 | -1.0572 |
| LST1 | 0.8716 | 2.8783 | 3.6270 | 4.13E-04 | 4.67E-02 | -1.0592 |
| VCX3A | -0.7798 | 0.2174 | -3.6048 | 4.47E-04 | 4.90E-02 | -1.1320 |
| A2M | 1.1362 | 7.0373 | 3.5924 | 4.66E-04 | 4.98E-02 | -1.1728 |
| UCK2 | -0.8471 | 2.0907 | -3.5866 | 4.76E-04 | 5.01E-02 | -1.1916 |
| AP1S2 | 0.6480 | 2.0814 | 3.5806 | 4.86E-04 | 5.07E-02 | -1.2114 |
| RUSC1-AS1 | -0.5622 | 2.0003 | -3.5780 | 4.90E-04 | 5.10E-02 | -1.2197 |
| SHROOM4 | 0.7212 | 1.6659 | 3.5777 | 4.91E-04 | 5.10E-02 | -1.2208 |
| FCGR2A | 0.7554 | 3.3453 | 3.5704 | 5.04E-04 | 5.19E-02 | -1.2447 |
| TMEM273 | 0.6196 | 1.7755 | 3.5645 | 5.14E-04 | 5.24E-02 | -1.2637 |
| ITM2B | 0.5929 | 5.6921 | 3.5553 | 5.31E-04 | 5.32E-02 | -1.2938 |
| DHRS3 | 0.8626 | 4.6813 | 3.5515 | 5.37E-04 | 5.35E-02 | -1.3060 |
| SYNE1 | 0.6094 | 1.6083 | 3.5473 | 5.45E-04 | 5.37E-02 | -1.3196 |
| RP1-159A19.4 | -0.5230 | 0.6018 | -3.5459 | 5.48E-04 | 5.39E-02 | -1.3242 |
| H2BC12 | -1.1614 | 6.1461 | -3.5444 | 5.51E-04 | 5.41E-02 | -1.3291 |
| PRRG4 | 0.6073 | 2.8420 | 3.5423 | 5.55E-04 | 5.42E-02 | -1.3357 |
| CYSRT1 | -0.6653 | 1.3587 | -3.5356 | 5.68E-04 | 5.50E-02 | -1.3573 |
| MIR210HG | -0.7136 | 2.2615 | -3.5341 | 5.71E-04 | 5.50E-02 | -1.3621 |
| NEXN | 0.6256 | 1.6919 | 3.5328 | 5.73E-04 | 5.52E-02 | -1.3664 |
| ADCY9 | 0.7098 | 2.3467 | 3.5261 | 5.87E-04 | 5.58E-02 | -1.3881 |
| FRZB | 0.9200 | 2.3939 | 3.5261 | 5.87E-04 | 5.58E-02 | -1.3882 |
| CTSO | 0.6463 | 4.0765 | 3.5241 | 5.91E-04 | 5.61E-02 | -1.3945 |
| TNFAIP8L2 | 0.7658 | 2.5511 | 3.5232 | 5.92E-04 | 5.61E-02 | -1.3973 |
| FCER1A | 1.3080 | 1.8986 | 3.5218 | 5.95E-04 | 5.62E-02 | -1.4020 |
| MEF2C | 0.6188 | 1.9380 | 3.5212 | 5.97E-04 | 5.62E-02 | -1.4040 |
| NR0B1 | -1.3528 | 0.6484 | -3.5150 | 6.10E-04 | 5.69E-02 | -1.4240 |
| SIGLEC9 | 0.5692 | 1.4674 | 3.5103 | 6.19E-04 | 5.71E-02 | -1.4388 |
| CTD-3025N20.3 | 0.5422 | 1.4576 | 3.5083 | 6.24E-04 | 5.73E-02 | -1.4453 |
| HHEX | 0.5729 | 2.1142 | 3.5038 | 6.33E-04 | 5.76E-02 | -1.4597 |
| EXPH5 | 0.5500 | 1.8834 | 3.5031 | 6.35E-04 | 5.76E-02 | -1.4622 |
| IL33 | 1.0685 | 2.6081 | 3.4970 | 6.48E-04 | 5.82E-02 | -1.4817 |
| SGMS2 | 0.6993 | 3.0591 | 3.4968 | 6.49E-04 | 5.82E-02 | -1.4821 |
| RP11-21L23.2 | -0.6146 | 0.6719 | -3.4954 | 6.52E-04 | 5.84E-02 | -1.4867 |
| NSG2 | -0.9303 | 0.2123 | -3.4897 | 6.65E-04 | 5.88E-02 | -1.5050 |
| LY6E | 1.2642 | 6.9526 | 3.4889 | 6.66E-04 | 5.88E-02 | -1.5076 |
| AC084117.3 | -0.5719 | 1.1781 | -3.4862 | 6.73E-04 | 5.92E-02 | -1.5162 |
| TRIL | 0.7827 | 1.4685 | 3.4824 | 6.81E-04 | 5.96E-02 | -1.5281 |
| CDK5RAP2 | -0.5184 | 2.8858 | -3.4769 | 6.94E-04 | 5.98E-02 | -1.5459 |
| PKD2 | 0.5465 | 2.8011 | 3.4759 | 6.97E-04 | 5.99E-02 | -1.5489 |
| PPIAP39 | 0.6979 | 1.4068 | 3.4702 | 7.10E-04 | 6.05E-02 | -1.5671 |
| MRPS6 | 0.5259 | 3.3943 | 3.4643 | 7.25E-04 | 6.12E-02 | -1.5859 |
| HNF1B | 0.8890 | 2.5734 | 3.4591 | 7.38E-04 | 6.19E-02 | -1.6025 |
| SHC1 | -0.5997 | 5.4539 | -3.4553 | 7.47E-04 | 6.21E-02 | -1.6145 |
| CSF1 | 0.7868 | 3.5983 | 3.4524 | 7.54E-04 | 6.25E-02 | -1.6235 |
| TBX5-AS1 | 0.5560 | 1.1136 | 3.4521 | 7.55E-04 | 6.25E-02 | -1.6245 |
| CKS1B | -0.7153 | 3.4007 | -3.4494 | 7.62E-04 | 6.27E-02 | -1.6330 |
| ELN | 1.2444 | 3.8298 | 3.4494 | 7.62E-04 | 6.27E-02 | -1.6330 |
| SPON1 | 0.9960 | 2.8943 | 3.4433 | 7.78E-04 | 6.33E-02 | -1.6526 |
| FCN1 | 0.8404 | 1.5766 | 3.4417 | 7.82E-04 | 6.35E-02 | -1.6575 |
| COA6 | -0.6082 | 4.1848 | -3.4402 | 7.86E-04 | 6.37E-02 | -1.6621 |
| CCDC8 | 0.7714 | 1.7369 | 3.4384 | 7.91E-04 | 6.39E-02 | -1.6680 |
| NXN | 0.7582 | 3.4528 | 3.4321 | 8.08E-04 | 6.45E-02 | -1.6877 |
| AXL | 0.7284 | 3.2760 | 3.4290 | 8.16E-04 | 6.49E-02 | -1.6976 |
| CYTH3 | 0.7634 | 3.3499 | 3.4266 | 8.23E-04 | 6.50E-02 | -1.7051 |
| MED27 | -0.5116 | 3.0796 | -3.4179 | 8.48E-04 | 6.62E-02 | -1.7326 |
| LBH | 0.8397 | 4.8932 | 3.4156 | 8.54E-04 | 6.63E-02 | -1.7397 |
| GPR34 | 0.7015 | 1.8339 | 3.4131 | 8.61E-04 | 6.66E-02 | -1.7477 |
| GLIS3 | 0.5988 | 1.4991 | 3.4130 | 8.62E-04 | 6.66E-02 | -1.7478 |
| RP11-21L23.3 | -0.5713 | 0.8502 | -3.4095 | 8.72E-04 | 6.70E-02 | -1.7589 |
| RP11-208G20.2 | -1.1103 | 0.9742 | -3.4059 | 8.82E-04 | 6.74E-02 | -1.7700 |
| ACSL5 | 1.1778 | 5.0494 | 3.4053 | 8.84E-04 | 6.74E-02 | -1.7719 |
| RBMS3 | 0.5309 | 1.2281 | 3.4044 | 8.87E-04 | 6.74E-02 | -1.7746 |
| RRM2B | 0.5422 | 3.2626 | 3.4035 | 8.89E-04 | 6.74E-02 | -1.7775 |
| FAM189A2 | 1.0011 | 2.0364 | 3.4009 | 8.97E-04 | 6.78E-02 | -1.7857 |
| RP11-470C13.4 | -0.6041 | 0.1271 | -3.4004 | 8.99E-04 | 6.78E-02 | -1.7873 |
| JAKMIP1 | -0.5426 | 0.7377 | -3.3970 | 9.09E-04 | 6.82E-02 | -1.7980 |
| ANTXR1 | 0.8946 | 3.8505 | 3.3970 | 9.09E-04 | 6.82E-02 | -1.7981 |
| MCRIP2 | -0.5243 | 3.0154 | -3.3914 | 9.26E-04 | 6.88E-02 | -1.8154 |
| RASL12 | 0.7036 | 2.3318 | 3.3895 | 9.32E-04 | 6.91E-02 | -1.8214 |
| LRP8 | -0.6050 | 1.4889 | -3.3843 | 9.49E-04 | 7.00E-02 | -1.8376 |
| RP11-575F12.3 | -0.5779 | 0.4237 | -3.3836 | 9.51E-04 | 7.01E-02 | -1.8397 |
| WFDC2 | 1.4104 | 7.5668 | 3.3820 | 9.56E-04 | 7.02E-02 | -1.8448 |
| GTPBP4 | -0.5640 | 3.3754 | -3.3790 | 9.65E-04 | 7.02E-02 | -1.8540 |
| DSP | -1.2357 | 4.7476 | -3.3760 | 9.75E-04 | 7.07E-02 | -1.8635 |
| RP11-575F12.2 | -0.5512 | 0.4205 | -3.3757 | 9.76E-04 | 7.07E-02 | -1.8644 |
| IL1R1 | 0.6417 | 4.2760 | 3.3662 | 1.01E-03 | 7.27E-02 | -1.8938 |
| IFNAR2 | 0.5223 | 2.9415 | 3.3643 | 1.01E-03 | 7.29E-02 | -1.8998 |
| SLAIN1 | 0.6356 | 2.1064 | 3.3635 | 1.02E-03 | 7.29E-02 | -1.9021 |
| RP11-284N8.3 | 0.9514 | 1.9140 | 3.3632 | 1.02E-03 | 7.29E-02 | -1.9033 |
| ITGB5 | 0.6113 | 4.5173 | 3.3612 | 1.02E-03 | 7.32E-02 | -1.9093 |
| ARHGAP20 | 0.6223 | 0.9980 | 3.3597 | 1.03E-03 | 7.32E-02 | -1.9139 |
| SYT5 | -0.5360 | 0.2738 | -3.3546 | 1.05E-03 | 7.38E-02 | -1.9298 |
| TREM2 | 0.9556 | 4.0607 | 3.3541 | 1.05E-03 | 7.39E-02 | -1.9314 |
| EVA1C | 0.7441 | 2.5243 | 3.3450 | 1.08E-03 | 7.50E-02 | -1.9593 |
| CCL2 | 0.9509 | 4.0665 | 3.3416 | 1.09E-03 | 7.55E-02 | -1.9700 |
| PLXND1 | 0.7333 | 4.4969 | 3.3385 | 1.10E-03 | 7.59E-02 | -1.9794 |
| RP11-750H9.5 | 0.5691 | 1.2495 | 3.3355 | 1.12E-03 | 7.66E-02 | -1.9888 |
| MSR1 | 0.8584 | 2.5401 | 3.3327 | 1.13E-03 | 7.70E-02 | -1.9974 |
| IL1B | 0.6328 | 1.4833 | 3.3316 | 1.13E-03 | 7.71E-02 | -2.0008 |
| GPC3 | 1.3408 | 2.7348 | 3.3297 | 1.14E-03 | 7.72E-02 | -2.0065 |
| HVCN1 | 0.5028 | 1.7754 | 3.3290 | 1.14E-03 | 7.72E-02 | -2.0087 |
| SELENOI | -0.5268 | 2.7623 | -3.3282 | 1.14E-03 | 7.73E-02 | -2.0111 |
| PLEKHG1 | 0.5923 | 2.1130 | 3.3260 | 1.15E-03 | 7.78E-02 | -2.0179 |
| CD4 | 0.8535 | 4.4003 | 3.3141 | 1.20E-03 | 7.98E-02 | -2.0544 |
| TMEM37 | 0.9280 | 3.0493 | 3.3135 | 1.20E-03 | 7.99E-02 | -2.0564 |
| H2BC17 | -0.5711 | 0.5827 | -3.3122 | 1.20E-03 | 7.99E-02 | -2.0601 |
| SESN3 | 0.7598 | 2.6247 | 3.3076 | 1.22E-03 | 8.01E-02 | -2.0743 |
| SLC34A2 | 1.4462 | 8.1896 | 3.3069 | 1.23E-03 | 8.01E-02 | -2.0763 |
| C1orf162 | 0.7720 | 2.9195 | 3.3059 | 1.23E-03 | 8.03E-02 | -2.0796 |
| RP11-115J16.1 | -0.8578 | 0.8465 | -3.2973 | 1.26E-03 | 8.12E-02 | -2.1056 |
| MAN1A1 | 0.7608 | 3.5858 | 3.2948 | 1.28E-03 | 8.15E-02 | -2.1134 |
| CH25H | 1.1383 | 2.3852 | 3.2946 | 1.28E-03 | 8.15E-02 | -2.1138 |
| CD1C | 0.9950 | 1.9102 | 3.2938 | 1.28E-03 | 8.16E-02 | -2.1163 |
| SCARF1 | 0.6868 | 2.4535 | 3.2929 | 1.28E-03 | 8.17E-02 | -2.1191 |
| SOD3 | 0.9896 | 4.1503 | 3.2913 | 1.29E-03 | 8.21E-02 | -2.1240 |
| PLEKHH2 | 0.7128 | 1.7521 | 3.2907 | 1.29E-03 | 8.21E-02 | -2.1259 |
| SNORD19 | -0.5460 | 1.5802 | -3.2901 | 1.30E-03 | 8.21E-02 | -2.1278 |
| HLA-DQB2 | 1.4228 | 4.5386 | 3.2900 | 1.30E-03 | 8.21E-02 | -2.1280 |
| FMO2 | 0.9632 | 2.2368 | 3.2884 | 1.30E-03 | 8.22E-02 | -2.1327 |
| RAB31 | 0.6865 | 4.3453 | 3.2818 | 1.33E-03 | 8.27E-02 | -2.1528 |
| C7 | 1.4170 | 3.3536 | 3.2779 | 1.35E-03 | 8.28E-02 | -2.1647 |
| SLC37A2 | 0.6347 | 1.7491 | 3.2767 | 1.35E-03 | 8.29E-02 | -2.1685 |
| ICAM1 | 1.0484 | 6.2441 | 3.2763 | 1.35E-03 | 8.29E-02 | -2.1695 |
| RP11-1148L6.8 | 0.5441 | 3.4971 | 3.2732 | 1.37E-03 | 8.35E-02 | -2.1789 |
| CX3CL1 | 1.2935 | 3.8101 | 3.2712 | 1.38E-03 | 8.37E-02 | -2.1849 |
| COX6CP1 | -1.1375 | 1.3849 | -3.2704 | 1.38E-03 | 8.37E-02 | -2.1874 |
| LINC01936 | 0.5791 | 0.8692 | 3.2622 | 1.42E-03 | 8.51E-02 | -2.2122 |
| SSX1 | -0.6977 | 0.2397 | -3.2582 | 1.44E-03 | 8.56E-02 | -2.2242 |
| DNER | -0.8855 | 0.9228 | -3.2582 | 1.44E-03 | 8.56E-02 | -2.2242 |
| FGL2 | 0.8775 | 2.9856 | 3.2582 | 1.44E-03 | 8.56E-02 | -2.2243 |
| IL1RAP | 0.5656 | 1.5533 | 3.2572 | 1.44E-03 | 8.56E-02 | -2.2274 |
| ITGA9 | 0.7979 | 2.2126 | 3.2563 | 1.45E-03 | 8.58E-02 | -2.2299 |
| MARCO | 1.5617 | 4.1359 | 3.2540 | 1.46E-03 | 8.59E-02 | -2.2369 |
| CLIC4 | 0.5527 | 4.9816 | 3.2538 | 1.46E-03 | 8.59E-02 | -2.2375 |
| PRKAR1B | -0.5938 | 2.5914 | -3.2532 | 1.46E-03 | 8.60E-02 | -2.2394 |
| OGDHL | -0.5456 | 0.4303 | -3.2506 | 1.47E-03 | 8.64E-02 | -2.2472 |
| LHFPL6 | 0.6233 | 3.6197 | 3.2475 | 1.49E-03 | 8.70E-02 | -2.2565 |
| SNORD14E | -0.8204 | 1.9431 | -3.2456 | 1.50E-03 | 8.73E-02 | -2.2623 |
| B4GALNT1 | -0.5870 | 0.5795 | -3.2431 | 1.51E-03 | 8.74E-02 | -2.2697 |
| RP11-452L6.1 | -0.5550 | 2.2643 | -3.2405 | 1.52E-03 | 8.79E-02 | -2.2775 |
| AMOTL2 | 0.6886 | 3.6002 | 3.2396 | 1.53E-03 | 8.81E-02 | -2.2802 |
| RP3-465N24.5 | -0.5034 | 0.8394 | -3.2332 | 1.56E-03 | 8.93E-02 | -2.2995 |
| SPI1 | 0.8350 | 4.2708 | 3.2308 | 1.57E-03 | 8.93E-02 | -2.3068 |
| TBX5 | 0.5752 | 1.4875 | 3.2302 | 1.57E-03 | 8.94E-02 | -2.3086 |
| EGLN3 | -1.2442 | 2.5497 | -3.2289 | 1.58E-03 | 8.94E-02 | -2.3124 |
| SAMD4A | 0.6848 | 1.7248 | 3.2282 | 1.58E-03 | 8.94E-02 | -2.3146 |
| TBX1 | 0.7565 | 1.3101 | 3.2267 | 1.59E-03 | 8.96E-02 | -2.3189 |
| ZFP36L1 | 0.7246 | 6.5133 | 3.2263 | 1.59E-03 | 8.96E-02 | -2.3203 |
| ADRB2 | 0.6249 | 1.2521 | 3.2228 | 1.61E-03 | 9.00E-02 | -2.3306 |
| TREML3P | -0.6022 | 0.5273 | -3.2221 | 1.62E-03 | 9.00E-02 | -2.3327 |
| PXDC1 | 0.7713 | 3.9493 | 3.2181 | 1.64E-03 | 9.04E-02 | -2.3448 |
| EGFL6 | 0.7250 | 2.5416 | 3.2177 | 1.64E-03 | 9.04E-02 | -2.3458 |
| PITX1 | -1.1846 | 1.8724 | -3.2169 | 1.64E-03 | 9.04E-02 | -2.3482 |
| PLN | 0.7297 | 1.8023 | 3.2166 | 1.64E-03 | 9.04E-02 | -2.3491 |
| LINC01833 | -0.6047 | 0.4478 | -3.2164 | 1.65E-03 | 9.04E-02 | -2.3497 |
| TMEM30B | 0.6085 | 4.1070 | 3.2164 | 1.65E-03 | 9.04E-02 | -2.3498 |
| RP3-512B11.3 | -0.6711 | 1.3411 | -3.2136 | 1.66E-03 | 9.09E-02 | -2.3581 |
| GLIPR2 | 0.8146 | 3.3951 | 3.2128 | 1.66E-03 | 9.10E-02 | -2.3606 |
| SLC7A1 | -0.5406 | 2.7165 | -3.2121 | 1.67E-03 | 9.11E-02 | -2.3626 |
| ADPRHL1 | -0.7240 | 1.6113 | -3.2104 | 1.68E-03 | 9.14E-02 | -2.3677 |
| PTPRE | 0.6785 | 2.6941 | 3.2098 | 1.68E-03 | 9.14E-02 | -2.3694 |
| CSF1R | 0.7833 | 3.7783 | 3.2076 | 1.69E-03 | 9.17E-02 | -2.3760 |
| SUCNR1 | 0.5268 | 0.9493 | 3.2073 | 1.69E-03 | 9.17E-02 | -2.3770 |
| CKS2 | -0.7947 | 5.0797 | -3.2068 | 1.70E-03 | 9.17E-02 | -2.3785 |
| APLP2 | 0.6465 | 7.1918 | 3.2055 | 1.70E-03 | 9.17E-02 | -2.3821 |
| LAMA2 | 0.5999 | 1.7639 | 3.2040 | 1.71E-03 | 9.21E-02 | -2.3869 |
| SNX18 | 0.5230 | 3.0579 | 3.2021 | 1.72E-03 | 9.21E-02 | -2.3923 |
| ITGAM | 0.7992 | 2.5391 | 3.2010 | 1.73E-03 | 9.23E-02 | -2.3957 |
| C1R | 0.7301 | 5.5244 | 3.2006 | 1.73E-03 | 9.23E-02 | -2.3969 |
| SCGN | -0.6631 | 0.2374 | -3.1995 | 1.74E-03 | 9.24E-02 | -2.4001 |
| SCG2 | -1.1993 | 1.0057 | -3.1972 | 1.75E-03 | 9.27E-02 | -2.4070 |
| ADGRF5 | 1.3107 | 4.6643 | 3.1937 | 1.77E-03 | 9.32E-02 | -2.4173 |
| MSMO1 | -0.5690 | 4.7497 | -3.1932 | 1.77E-03 | 9.32E-02 | -2.4189 |
| LINC02313 | -0.5353 | 0.3798 | -3.1880 | 1.80E-03 | 9.42E-02 | -2.4342 |
| ARVCF | 0.5820 | 2.5462 | 3.1875 | 1.81E-03 | 9.42E-02 | -2.4357 |
| GGTA1 | 0.5297 | 1.3906 | 3.1860 | 1.81E-03 | 9.45E-02 | -2.4401 |
| SYNGR4 | -0.5401 | 0.4455 | -3.1782 | 1.86E-03 | 9.56E-02 | -2.4632 |
| RPRM | -0.6836 | 0.5649 | -3.1726 | 1.89E-03 | 9.64E-02 | -2.4798 |
| RPP25 | -0.5796 | 2.6469 | -3.1725 | 1.89E-03 | 9.64E-02 | -2.4799 |
| ERG | 0.5163 | 1.5659 | 3.1707 | 1.90E-03 | 9.67E-02 | -2.4854 |
| PLTP | 0.9156 | 5.5410 | 3.1701 | 1.91E-03 | 9.68E-02 | -2.4871 |
| CDC20B | -0.6786 | 0.3481 | -3.1679 | 1.92E-03 | 9.71E-02 | -2.4937 |
| RP11-147L13.11 | 0.6190 | 4.0730 | 3.1667 | 1.93E-03 | 9.72E-02 | -2.4972 |
| LMO3 | 1.2277 | 3.0980 | 3.1649 | 1.94E-03 | 9.73E-02 | -2.5026 |
| FGFR2 | 0.8059 | 1.8567 | 3.1610 | 1.96E-03 | 9.79E-02 | -2.5138 |
| TNS1 | 0.8081 | 3.9285 | 3.1593 | 1.97E-03 | 9.79E-02 | -2.5188 |
| ETV1 | 0.8583 | 2.0661 | 3.1592 | 1.98E-03 | 9.79E-02 | -2.5191 |
| IER5 | -0.6561 | 3.9138 | -3.1592 | 1.98E-03 | 9.79E-02 | -2.5193 |
| PODN | 0.8627 | 3.1071 | 3.1587 | 1.98E-03 | 9.79E-02 | -2.5207 |
| TIMM8AP1 | 1.1937 | 2.0085 | 3.1524 | 2.02E-03 | 9.89E-02 | -2.5393 |
| LINC01816 | -0.5147 | 1.0739 | -3.1522 | 2.02E-03 | 9.89E-02 | -2.5399 |
| CD47 | 0.6102 | 4.8428 | 3.1518 | 2.02E-03 | 9.89E-02 | -2.5409 |
| MAFG | -0.5249 | 3.2907 | -3.1501 | 2.03E-03 | 9.91E-02 | -2.5459 |
| LMO2 | 0.5637 | 2.1378 | 3.1489 | 2.04E-03 | 9.93E-02 | -2.5496 |
| ITGA8 | 0.6889 | 1.5454 | 3.1473 | 2.05E-03 | 9.95E-02 | -2.5542 |
| SNED1 | 0.5412 | 1.9147 | 3.1455 | 2.06E-03 | 9.99E-02 | -2.5595 |
| INSL4 | -1.2642 | 0.6350 | -3.1436 | 2.08E-03 | 1.00E-01 | -2.5651 |
| SYT4 | -0.5553 | 0.1436 | -3.1414 | 2.09E-03 | 1.00E-01 | -2.5715 |
| MCEMP1 | 1.2063 | 1.9100 | 3.1326 | 2.15E-03 | 1.02E-01 | -2.5971 |
| ST6GALNAC1 | 1.1595 | 3.8249 | 3.1322 | 2.15E-03 | 1.02E-01 | -2.5982 |
| PDP1 | 0.5919 | 3.0539 | 3.1301 | 2.17E-03 | 1.02E-01 | -2.6043 |
| MMP2 | 0.9979 | 5.8460 | 3.1287 | 2.18E-03 | 1.02E-01 | -2.6085 |
| INAFM2 | 0.5636 | 4.0270 | 3.1181 | 2.25E-03 | 1.04E-01 | -2.6392 |
| MAMDC2 | 0.8963 | 1.4827 | 3.1167 | 2.26E-03 | 1.04E-01 | -2.6434 |
| VSIG4 | 1.0166 | 3.8722 | 3.1152 | 2.27E-03 | 1.04E-01 | -2.6478 |
| GPNMB | 0.9609 | 5.1322 | 3.1119 | 2.29E-03 | 1.05E-01 | -2.6575 |
| RFTN1 | 0.7291 | 3.8316 | 3.1103 | 2.31E-03 | 1.05E-01 | -2.6620 |
| DES | 1.1071 | 2.0035 | 3.1096 | 2.31E-03 | 1.05E-01 | -2.6640 |
| TLE2 | 0.7442 | 3.7561 | 3.1082 | 2.32E-03 | 1.05E-01 | -2.6682 |
| MAGEC1 | -0.6787 | 0.2174 | -3.1054 | 2.34E-03 | 1.06E-01 | -2.6762 |
| OPN3 | -0.9366 | 2.4694 | -3.1046 | 2.35E-03 | 1.06E-01 | -2.6786 |
| CITF22-49E9.3 | -0.5950 | 1.2963 | -3.1021 | 2.37E-03 | 1.06E-01 | -2.6858 |
| SGCA | 0.6719 | 1.3757 | 3.1018 | 2.37E-03 | 1.06E-01 | -2.6867 |
| BHLHA15 | -0.8756 | 1.8979 | -3.0992 | 2.39E-03 | 1.06E-01 | -2.6942 |
| KYNU | -0.9148 | 1.1014 | -3.0991 | 2.39E-03 | 1.06E-01 | -2.6945 |
| SPRED1 | 0.6080 | 3.1308 | 3.0977 | 2.40E-03 | 1.06E-01 | -2.6986 |
| CSDC2 | 0.5082 | 1.2421 | 3.0960 | 2.41E-03 | 1.06E-01 | -2.7035 |
| FAS | 0.6829 | 2.5898 | 3.0933 | 2.43E-03 | 1.07E-01 | -2.7113 |
| TXN | -0.6991 | 7.2544 | -3.0928 | 2.44E-03 | 1.07E-01 | -2.7126 |
| RAB42 | 0.6092 | 1.9424 | 3.0920 | 2.44E-03 | 1.07E-01 | -2.7151 |
| PDE9A | 0.6322 | 2.3237 | 3.0912 | 2.45E-03 | 1.07E-01 | -2.7172 |
| ESYT3 | 0.7221 | 1.5012 | 3.0906 | 2.45E-03 | 1.07E-01 | -2.7191 |
| CASS4 | 0.5104 | 1.2473 | 3.0898 | 2.46E-03 | 1.07E-01 | -2.7215 |
| FBXO6 | 0.5262 | 3.8626 | 3.0886 | 2.47E-03 | 1.07E-01 | -2.7248 |
| ID4 | 1.0208 | 2.8122 | 3.0879 | 2.47E-03 | 1.08E-01 | -2.7269 |
| DHCR7 | -0.5764 | 4.2160 | -3.0873 | 2.48E-03 | 1.08E-01 | -2.7286 |
| MTND4P24 | -1.0576 | 0.8785 | -3.0866 | 2.48E-03 | 1.08E-01 | -2.7306 |
| HSPD1 | -0.5491 | 6.0643 | -3.0864 | 2.49E-03 | 1.08E-01 | -2.7310 |
| IFT57 | 0.8847 | 4.2449 | 3.0860 | 2.49E-03 | 1.08E-01 | -2.7322 |
| EPB41L2 | 0.5769 | 2.1796 | 3.0831 | 2.51E-03 | 1.08E-01 | -2.7407 |
| DOK2 | 0.8044 | 2.9656 | 3.0804 | 2.53E-03 | 1.08E-01 | -2.7485 |
| LPCAT2 | 0.5085 | 2.0385 | 3.0790 | 2.54E-03 | 1.08E-01 | -2.7524 |
| LY86 | 0.7850 | 2.8229 | 3.0767 | 2.56E-03 | 1.09E-01 | -2.7590 |
| HILPDA | -0.9407 | 3.3177 | -3.0751 | 2.57E-03 | 1.09E-01 | -2.7636 |
| PTGER1 | 0.5015 | 0.7636 | 3.0713 | 2.61E-03 | 1.10E-01 | -2.7746 |
| MTCO1P40 | -1.7734 | 2.3324 | -3.0709 | 2.61E-03 | 1.10E-01 | -2.7757 |
| B4GALNT3 | 0.9572 | 3.5069 | 3.0707 | 2.61E-03 | 1.10E-01 | -2.7763 |
| PIM3 | -0.5717 | 5.8120 | -3.0707 | 2.61E-03 | 1.10E-01 | -2.7763 |
| VSIG2 | 1.3502 | 3.5817 | 3.0688 | 2.63E-03 | 1.10E-01 | -2.7817 |
| DAB2 | 0.6412 | 3.0513 | 3.0676 | 2.64E-03 | 1.10E-01 | -2.7851 |
| SNX25 | 0.6920 | 3.0141 | 3.0660 | 2.65E-03 | 1.10E-01 | -2.7898 |
| FXYD6 | 0.5784 | 1.8907 | 3.0657 | 2.65E-03 | 1.10E-01 | -2.7905 |
| TGFBR2 | 0.7282 | 5.0268 | 3.0654 | 2.65E-03 | 1.10E-01 | -2.7916 |
| IL6ST | 0.6388 | 4.3784 | 3.0632 | 2.67E-03 | 1.10E-01 | -2.7978 |
| MGP | 0.9496 | 5.8866 | 3.0586 | 2.71E-03 | 1.11E-01 | -2.8109 |
| OSBPL1A | 0.5150 | 2.3470 | 3.0575 | 2.72E-03 | 1.12E-01 | -2.8142 |
| ARHGDIB | 0.6884 | 6.7003 | 3.0551 | 2.74E-03 | 1.12E-01 | -2.8210 |
| SUSD2 | 1.6571 | 4.2455 | 3.0543 | 2.75E-03 | 1.12E-01 | -2.8231 |
| SOX12 | -0.5893 | 3.1360 | -3.0539 | 2.75E-03 | 1.12E-01 | -2.8243 |
| CHRDL1 | 1.0901 | 2.0673 | 3.0523 | 2.76E-03 | 1.12E-01 | -2.8289 |
| H2BC11 | -0.8458 | 1.3418 | -3.0516 | 2.77E-03 | 1.12E-01 | -2.8309 |
| INMT | 1.1633 | 2.5768 | 3.0472 | 2.81E-03 | 1.13E-01 | -2.8434 |
| SLC15A3 | 0.7175 | 3.1161 | 3.0455 | 2.82E-03 | 1.13E-01 | -2.8483 |
| SLCO2A1 | 1.0093 | 3.4782 | 3.0446 | 2.83E-03 | 1.13E-01 | -2.8510 |
| RP11-21L23.4 | -0.6539 | 0.5355 | -3.0443 | 2.83E-03 | 1.13E-01 | -2.8519 |
| OSCAR | 0.8350 | 2.6682 | 3.0441 | 2.84E-03 | 1.13E-01 | -2.8522 |
| MRC1 | 1.1290 | 3.4567 | 3.0417 | 2.86E-03 | 1.13E-01 | -2.8590 |
| CD1A | 1.1861 | 1.6769 | 3.0410 | 2.86E-03 | 1.13E-01 | -2.8610 |
| SLC22A3 | 1.1127 | 3.0678 | 3.0382 | 2.89E-03 | 1.14E-01 | -2.8691 |
| PTK7 | 0.7019 | 4.4287 | 3.0366 | 2.90E-03 | 1.14E-01 | -2.8737 |
| NUPR1 | 0.8055 | 3.9107 | 3.0336 | 2.93E-03 | 1.15E-01 | -2.8822 |
| STAC | 0.6510 | 1.1683 | 3.0310 | 2.95E-03 | 1.15E-01 | -2.8894 |
| AIF1 | 0.7805 | 4.3618 | 3.0275 | 2.98E-03 | 1.15E-01 | -2.8995 |
| HIP1 | 0.7154 | 3.4005 | 3.0266 | 2.99E-03 | 1.16E-01 | -2.9020 |
| HPGDS | 0.6624 | 1.3637 | 3.0262 | 3.00E-03 | 1.16E-01 | -2.9030 |
| CD300C | 0.6042 | 1.5593 | 3.0248 | 3.01E-03 | 1.16E-01 | -2.9070 |
| CD302 | 0.6299 | 1.9465 | 3.0213 | 3.04E-03 | 1.16E-01 | -2.9169 |
| ARHGAP29 | 0.5529 | 1.9712 | 3.0198 | 3.06E-03 | 1.17E-01 | -2.9212 |
| FBLN1 | 0.9478 | 4.2370 | 3.0193 | 3.06E-03 | 1.17E-01 | -2.9228 |
| SVEP1 | 0.6117 | 1.1472 | 3.0175 | 3.08E-03 | 1.17E-01 | -2.9278 |
| CD207 | 1.3453 | 1.7955 | 3.0157 | 3.10E-03 | 1.17E-01 | -2.9328 |
| VTN | -0.6775 | 0.6166 | -3.0142 | 3.11E-03 | 1.17E-01 | -2.9370 |
| P2RY13 | 0.6582 | 1.6420 | 3.0140 | 3.11E-03 | 1.17E-01 | -2.9375 |
| MS4A7 | 0.8054 | 2.8583 | 3.0121 | 3.13E-03 | 1.18E-01 | -2.9431 |
| GPC4 | 0.9271 | 4.4413 | 3.0089 | 3.16E-03 | 1.18E-01 | -2.9520 |
| HDHD3 | -0.5166 | 4.1321 | -3.0081 | 3.17E-03 | 1.19E-01 | -2.9543 |
| RAB32 | 0.5939 | 3.9224 | 3.0063 | 3.19E-03 | 1.19E-01 | -2.9594 |
| ZNF608 | 0.5433 | 1.9947 | 3.0061 | 3.19E-03 | 1.19E-01 | -2.9598 |
| BMP4 | 0.9498 | 2.2913 | 3.0050 | 3.20E-03 | 1.19E-01 | -2.9630 |
| FBLN5 | 0.7565 | 3.0569 | 3.0032 | 3.22E-03 | 1.19E-01 | -2.9680 |
| PAQR6 | -0.6810 | 1.6071 | -3.0025 | 3.22E-03 | 1.19E-01 | -2.9701 |
| ARHGEF6 | 0.6012 | 2.1788 | 3.0012 | 3.24E-03 | 1.19E-01 | -2.9738 |
| TBX4 | 0.7411 | 1.6398 | 3.0008 | 3.24E-03 | 1.19E-01 | -2.9749 |
| KCNA3 | 0.6428 | 1.3165 | 2.9985 | 3.26E-03 | 1.19E-01 | -2.9814 |
| MPEG1 | 0.7778 | 3.2495 | 2.9975 | 3.27E-03 | 1.19E-01 | -2.9841 |
| LCP1 | 0.8684 | 5.0182 | 2.9935 | 3.31E-03 | 1.20E-01 | -2.9953 |
| H4C9 | -0.8380 | 3.3875 | -2.9933 | 3.32E-03 | 1.20E-01 | -2.9959 |
| CX3CR1 | 0.6227 | 1.0464 | 2.9926 | 3.32E-03 | 1.20E-01 | -2.9979 |
| MFNG | 0.5428 | 2.7272 | 2.9912 | 3.34E-03 | 1.21E-01 | -3.0017 |
| SNAP25 | -0.6487 | 0.6453 | -2.9889 | 3.36E-03 | 1.21E-01 | -3.0082 |
| ZC2HC1A | 0.5635 | 2.2553 | 2.9851 | 3.40E-03 | 1.22E-01 | -3.0188 |
| PCP4L1 | 1.6461 | 3.1144 | 2.9844 | 3.41E-03 | 1.22E-01 | -3.0208 |
| USP44 | 0.5323 | 0.9930 | 2.9803 | 3.45E-03 | 1.23E-01 | -3.0324 |
| PAMR1 | 0.5036 | 1.1009 | 2.9801 | 3.45E-03 | 1.23E-01 | -3.0330 |
| GRK3 | 0.6034 | 2.1537 | 2.9752 | 3.50E-03 | 1.23E-01 | -3.0465 |
| CACHD1 | 0.8441 | 1.9361 | 2.9726 | 3.53E-03 | 1.23E-01 | -3.0539 |
| SNORD69 | -0.5669 | 1.5112 | -2.9714 | 3.54E-03 | 1.23E-01 | -3.0572 |
| SSC5D | 0.6516 | 2.0107 | 2.9708 | 3.55E-03 | 1.23E-01 | -3.0588 |
| SNTB1 | 0.7784 | 3.4765 | 2.9705 | 3.55E-03 | 1.23E-01 | -3.0597 |
| FGD6 | 0.5773 | 2.6605 | 2.9697 | 3.56E-03 | 1.23E-01 | -3.0620 |
| CLIC6 | 1.4145 | 4.7853 | 2.9678 | 3.58E-03 | 1.23E-01 | -3.0673 |
| FCGBP | 1.1468 | 2.2611 | 2.9662 | 3.60E-03 | 1.23E-01 | -3.0715 |
| MLXP1 | -0.6437 | 0.3274 | -2.9658 | 3.60E-03 | 1.23E-01 | -3.0727 |
| CD1E | 0.7952 | 1.2730 | 2.9658 | 3.61E-03 | 1.23E-01 | -3.0728 |
| MTND1P23 | -2.1827 | 2.3665 | -2.9651 | 3.61E-03 | 1.23E-01 | -3.0748 |
| STING1 | 0.6838 | 4.8647 | 2.9645 | 3.62E-03 | 1.23E-01 | -3.0764 |
| FMO3 | 0.6033 | 1.7200 | 2.9620 | 3.65E-03 | 1.23E-01 | -3.0834 |
| DTL | -0.5539 | 1.8702 | -2.9614 | 3.65E-03 | 1.23E-01 | -3.0851 |
| S100B | 1.0365 | 2.4113 | 2.9602 | 3.67E-03 | 1.23E-01 | -3.0883 |
| SLC47A1 | 0.8167 | 1.5360 | 2.9563 | 3.71E-03 | 1.23E-01 | -3.0992 |
| C4A | 0.7442 | 1.7424 | 2.9562 | 3.71E-03 | 1.23E-01 | -3.0995 |
| LMO7-AS1 | 0.5380 | 1.1369 | 2.9561 | 3.71E-03 | 1.23E-01 | -3.0999 |
| IFIT2 | 0.6826 | 2.6397 | 2.9556 | 3.72E-03 | 1.23E-01 | -3.1011 |
| ECM2 | 0.5062 | 1.5006 | 2.9551 | 3.72E-03 | 1.23E-01 | -3.1025 |
| OGFRL1 | 0.5514 | 2.3893 | 2.9531 | 3.75E-03 | 1.23E-01 | -3.1080 |
| MYH10 | 0.5718 | 3.6006 | 2.9492 | 3.79E-03 | 1.23E-01 | -3.1187 |
| COL21A1 | 0.7215 | 1.3254 | 2.9490 | 3.79E-03 | 1.23E-01 | -3.1194 |
| IGSF6 | 0.7406 | 2.6702 | 2.9470 | 3.82E-03 | 1.23E-01 | -3.1248 |
| CHGA | -0.9119 | 0.3436 | -2.9456 | 3.83E-03 | 1.23E-01 | -3.1289 |
| DPYSL2 | 0.7597 | 4.3305 | 2.9452 | 3.84E-03 | 1.23E-01 | -3.1300 |
| PKMYT1 | -0.6212 | 1.7400 | -2.9421 | 3.87E-03 | 1.23E-01 | -3.1384 |
| TYROBP | 0.8027 | 6.1007 | 2.9411 | 3.89E-03 | 1.23E-01 | -3.1414 |
| TXNRD1 | -1.2714 | 5.0449 | -2.9397 | 3.90E-03 | 1.23E-01 | -3.1451 |
| CXCL14 | 1.8341 | 4.1184 | 2.9369 | 3.94E-03 | 1.23E-01 | -3.1530 |
| HMCN1 | 0.6086 | 1.3509 | 2.9365 | 3.94E-03 | 1.23E-01 | -3.1538 |
| PAX8-AS1 | 0.6109 | 0.8865 | 2.9354 | 3.95E-03 | 1.23E-01 | -3.1570 |
| LINC00674 | 0.5551 | 2.8662 | 2.9334 | 3.98E-03 | 1.23E-01 | -3.1626 |
| ENPP2 | 0.6727 | 2.9511 | 2.9311 | 4.00E-03 | 1.23E-01 | -3.1690 |
| COX7A1 | 0.5253 | 2.4224 | 2.9310 | 4.01E-03 | 1.23E-01 | -3.1692 |
| PTGDS | 1.2065 | 4.6069 | 2.9301 | 4.02E-03 | 1.23E-01 | -3.1717 |
| TMEM125 | 0.7489 | 5.4670 | 2.9292 | 4.03E-03 | 1.23E-01 | -3.1741 |
| SNORD19C | -0.6171 | 2.2736 | -2.9266 | 4.06E-03 | 1.24E-01 | -3.1812 |
| NRGN | 1.2489 | 4.1870 | 2.9257 | 4.07E-03 | 1.24E-01 | -3.1838 |
| SRI | 0.5053 | 4.7057 | 2.9256 | 4.07E-03 | 1.24E-01 | -3.1841 |
| SCARF2 | 0.5988 | 2.1468 | 2.9243 | 4.09E-03 | 1.24E-01 | -3.1876 |
| RENBP | 0.7461 | 3.3249 | 2.9228 | 4.11E-03 | 1.24E-01 | -3.1917 |
| PIGR | 2.0635 | 6.2221 | 2.9219 | 4.12E-03 | 1.24E-01 | -3.1941 |
| ARHGAP23 | 0.5250 | 2.5371 | 2.9209 | 4.13E-03 | 1.24E-01 | -3.1969 |
| HSPE1 | -0.5092 | 5.3150 | -2.9202 | 4.14E-03 | 1.24E-01 | -3.1988 |
| GXYLT2 | 0.5520 | 1.5258 | 2.9201 | 4.14E-03 | 1.24E-01 | -3.1992 |
| SLC38A3 | -0.6485 | 0.3842 | -2.9200 | 4.14E-03 | 1.24E-01 | -3.1994 |
| EBF4 | 0.7014 | 2.3052 | 2.9193 | 4.15E-03 | 1.24E-01 | -3.2013 |
| NRROS | 0.5314 | 1.9440 | 2.9166 | 4.18E-03 | 1.24E-01 | -3.2087 |
| MS4A4A | 0.7302 | 2.8595 | 2.9137 | 4.22E-03 | 1.25E-01 | -3.2166 |
| NEDD9 | 0.8039 | 3.4532 | 2.9091 | 4.28E-03 | 1.25E-01 | -3.2293 |
| GABRB3 | -0.5887 | 0.6186 | -2.9090 | 4.28E-03 | 1.25E-01 | -3.2295 |
| HLF | 0.9780 | 1.8223 | 2.9079 | 4.29E-03 | 1.25E-01 | -3.2325 |
| NME4 | -0.5629 | 4.7169 | -2.9074 | 4.30E-03 | 1.25E-01 | -3.2339 |
| COL9A2 | 1.0295 | 3.1010 | 2.9068 | 4.31E-03 | 1.25E-01 | -3.2357 |
| CD83 | 0.6841 | 3.4913 | 2.9054 | 4.33E-03 | 1.25E-01 | -3.2395 |
| ENG | 0.6410 | 5.2396 | 2.9043 | 4.34E-03 | 1.25E-01 | -3.2423 |
| EFS | 0.9374 | 2.6540 | 2.9027 | 4.36E-03 | 1.25E-01 | -3.2469 |
| HOXA5 | 0.5347 | 1.9021 | 2.9015 | 4.38E-03 | 1.25E-01 | -3.2501 |
| CD52 | 0.9368 | 5.0586 | 2.9012 | 4.38E-03 | 1.25E-01 | -3.2510 |
| KHDRBS2 | 0.6513 | 0.7846 | 2.9003 | 4.39E-03 | 1.25E-01 | -3.2532 |
| RP11-111M22.3 | -0.5638 | 2.2099 | -2.8987 | 4.41E-03 | 1.25E-01 | -3.2576 |
| ANKRD65 | 1.0313 | 3.6240 | 2.8984 | 4.42E-03 | 1.25E-01 | -3.2586 |
| B3GNT8 | 0.9511 | 3.3526 | 2.8973 | 4.43E-03 | 1.25E-01 | -3.2614 |
| RBPMS | 0.6101 | 3.5146 | 2.8964 | 4.44E-03 | 1.25E-01 | -3.2639 |
| B4GALT4 | -0.5035 | 2.4332 | -2.8951 | 4.46E-03 | 1.26E-01 | -3.2674 |
| RP11-286N22.16 | -0.5654 | 0.7892 | -2.8933 | 4.48E-03 | 1.26E-01 | -3.2723 |
| CCDC3 | 0.6327 | 2.3001 | 2.8921 | 4.50E-03 | 1.26E-01 | -3.2758 |
| GRAMD2B | 0.5006 | 2.9308 | 2.8891 | 4.54E-03 | 1.27E-01 | -3.2838 |
| GPI | -0.5515 | 5.2905 | -2.8857 | 4.59E-03 | 1.28E-01 | -3.2931 |
| XXbac-BPG181B23.7 | 0.5235 | 1.5054 | 2.8817 | 4.64E-03 | 1.29E-01 | -3.3039 |
| SHISA3 | 1.5889 | 1.8116 | 2.8810 | 4.65E-03 | 1.29E-01 | -3.3058 |
| PRAM1 | 0.5614 | 1.4621 | 2.8780 | 4.69E-03 | 1.29E-01 | -3.3141 |
| CYP2S1 | 0.9128 | 2.7484 | 2.8759 | 4.72E-03 | 1.30E-01 | -3.3198 |
| ST3GAL5 | 0.9126 | 3.8465 | 2.8758 | 4.72E-03 | 1.30E-01 | -3.3200 |
| PRC1 | -0.6269 | 2.4806 | -2.8744 | 4.74E-03 | 1.30E-01 | -3.3238 |
| ARHGAP18 | 0.5112 | 3.3181 | 2.8740 | 4.75E-03 | 1.30E-01 | -3.3249 |
| TRPV2 | 0.6188 | 2.9306 | 2.8682 | 4.83E-03 | 1.31E-01 | -3.3407 |
| RP1-86C11.7 | -0.6286 | 0.9088 | -2.8659 | 4.86E-03 | 1.32E-01 | -3.3466 |
| GAS7 | 0.5750 | 1.9850 | 2.8631 | 4.91E-03 | 1.32E-01 | -3.3543 |
| NBL1 | 0.7459 | 4.2930 | 2.8627 | 4.91E-03 | 1.32E-01 | -3.3555 |
| TRIM9 | -0.5028 | 0.3750 | -2.8616 | 4.93E-03 | 1.32E-01 | -3.3584 |
| PTGER4 | 0.6623 | 2.0253 | 2.8614 | 4.93E-03 | 1.32E-01 | -3.3589 |
| COL8A1 | 0.8697 | 3.6446 | 2.8590 | 4.97E-03 | 1.33E-01 | -3.3654 |
| MAL | 0.8907 | 2.0189 | 2.8576 | 4.99E-03 | 1.33E-01 | -3.3693 |
| CYBB | 0.9210 | 4.1409 | 2.8570 | 5.00E-03 | 1.33E-01 | -3.3708 |
| LMOD1 | 0.6757 | 2.2645 | 2.8563 | 5.01E-03 | 1.33E-01 | -3.3727 |
| CABLES1 | 0.6812 | 2.5216 | 2.8560 | 5.01E-03 | 1.33E-01 | -3.3736 |
| MAL2 | 0.6575 | 6.6742 | 2.8556 | 5.02E-03 | 1.33E-01 | -3.3745 |
| ABCB6 | -0.5261 | 1.5801 | -2.8544 | 5.03E-03 | 1.33E-01 | -3.3777 |
| SORBS1 | 0.5015 | 1.6378 | 2.8534 | 5.05E-03 | 1.33E-01 | -3.3805 |
| EGR2 | 0.6329 | 2.2191 | 2.8531 | 5.05E-03 | 1.33E-01 | -3.3812 |
| CAT | 0.6123 | 4.6517 | 2.8530 | 5.05E-03 | 1.33E-01 | -3.3815 |
| HHIP-AS1 | 0.8938 | 1.3412 | 2.8497 | 5.10E-03 | 1.34E-01 | -3.3905 |
| NKX2-1 | 1.2092 | 5.6542 | 2.8485 | 5.12E-03 | 1.34E-01 | -3.3936 |
| TMEM130 | 1.0776 | 1.8086 | 2.8485 | 5.12E-03 | 1.34E-01 | -3.3937 |
| CLEC10A | 0.7048 | 1.9430 | 2.8460 | 5.16E-03 | 1.34E-01 | -3.4004 |
| MAGI3 | 0.6311 | 2.6104 | 2.8441 | 5.19E-03 | 1.34E-01 | -3.4056 |
| BICC1 | 0.5992 | 1.6038 | 2.8440 | 5.19E-03 | 1.34E-01 | -3.4057 |
| PTGIS | 0.7314 | 2.0610 | 2.8436 | 5.20E-03 | 1.34E-01 | -3.4068 |
| SPRY1 | 0.5239 | 3.5466 | 2.8417 | 5.23E-03 | 1.34E-01 | -3.4119 |
| FABP3 | 1.2084 | 3.5213 | 2.8415 | 5.23E-03 | 1.34E-01 | -3.4124 |
| MPZL2 | 0.7440 | 4.9314 | 2.8414 | 5.23E-03 | 1.34E-01 | -3.4127 |
| IQCD | -0.6362 | 1.7731 | -2.8396 | 5.26E-03 | 1.34E-01 | -3.4176 |
| MYH11 | 0.8867 | 2.5189 | 2.8369 | 5.30E-03 | 1.34E-01 | -3.4247 |
| CMTM7 | 0.5141 | 3.1377 | 2.8358 | 5.32E-03 | 1.34E-01 | -3.4277 |
| VSTM4 | 0.5126 | 1.5439 | 2.8356 | 5.32E-03 | 1.34E-01 | -3.4282 |
| CYCSP6 | -0.6605 | 0.4680 | -2.8353 | 5.33E-03 | 1.34E-01 | -3.4290 |
| SFTPA2 | 2.2822 | 8.6275 | 2.8349 | 5.33E-03 | 1.34E-01 | -3.4302 |
| PELATON | 0.7065 | 2.1753 | 2.8344 | 5.34E-03 | 1.34E-01 | -3.4314 |
| HROB | -0.5695 | 1.6047 | -2.8340 | 5.35E-03 | 1.34E-01 | -3.4324 |
| PTGS1 | 0.5083 | 2.0350 | 2.8322 | 5.37E-03 | 1.35E-01 | -3.4374 |
| SFTPA1 | 2.3335 | 8.3356 | 2.8321 | 5.38E-03 | 1.35E-01 | -3.4375 |
| CEBPA | 0.8858 | 3.0659 | 2.8318 | 5.38E-03 | 1.35E-01 | -3.4385 |
| MT-TM | -0.9039 | 1.1952 | -2.8292 | 5.42E-03 | 1.35E-01 | -3.4453 |
| SMIM3 | 0.5881 | 3.3476 | 2.8284 | 5.44E-03 | 1.35E-01 | -3.4476 |
| PTPN22 | 0.5480 | 1.6839 | 2.8278 | 5.44E-03 | 1.35E-01 | -3.4491 |
| H3C10 | -0.9357 | 2.0321 | -2.8266 | 5.46E-03 | 1.35E-01 | -3.4524 |
| CILP2 | 0.8014 | 1.6804 | 2.8265 | 5.46E-03 | 1.35E-01 | -3.4525 |
| DSG2 | -0.8490 | 4.5868 | -2.8251 | 5.49E-03 | 1.35E-01 | -3.4563 |
| PILRA | 0.5939 | 2.5088 | 2.8240 | 5.51E-03 | 1.35E-01 | -3.4592 |
| UBTD1 | 0.5607 | 4.2648 | 2.8220 | 5.54E-03 | 1.36E-01 | -3.4646 |
| MUC21 | 1.6283 | 3.3664 | 2.8172 | 5.62E-03 | 1.36E-01 | -3.4774 |
| LIPA | 0.6413 | 4.6633 | 2.8154 | 5.65E-03 | 1.37E-01 | -3.4821 |
| LIX1L | 0.5237 | 3.1360 | 2.8140 | 5.67E-03 | 1.37E-01 | -3.4859 |
| ENPP5 | 0.6504 | 2.8286 | 2.8055 | 5.81E-03 | 1.38E-01 | -3.5084 |
| HK3 | 0.7987 | 2.4736 | 2.8041 | 5.84E-03 | 1.38E-01 | -3.5121 |
| PLLP | 0.6198 | 2.0974 | 2.8040 | 5.84E-03 | 1.38E-01 | -3.5125 |
| LRRC25 | 0.5601 | 2.1817 | 2.8028 | 5.86E-03 | 1.39E-01 | -3.5156 |
| H2BC4 | -1.0322 | 2.8667 | -2.8009 | 5.89E-03 | 1.39E-01 | -3.5206 |
| INA | -0.7452 | 0.4908 | -2.8005 | 5.90E-03 | 1.39E-01 | -3.5217 |
| IFIT1 | 0.9218 | 2.6512 | 2.7987 | 5.93E-03 | 1.39E-01 | -3.5265 |
| MYL9 | 0.7070 | 5.6615 | 2.7984 | 5.93E-03 | 1.39E-01 | -3.5273 |
| CD86 | 0.5643 | 2.4395 | 2.7979 | 5.94E-03 | 1.39E-01 | -3.5286 |
| GPD1 | 0.6583 | 1.0409 | 2.7968 | 5.96E-03 | 1.40E-01 | -3.5315 |
| ACE | 0.5949 | 2.5338 | 2.7962 | 5.97E-03 | 1.40E-01 | -3.5331 |
| RNY4P19 | -0.5881 | 0.7131 | -2.7959 | 5.98E-03 | 1.40E-01 | -3.5337 |
| CALCRL | 0.6400 | 2.2715 | 2.7951 | 5.99E-03 | 1.40E-01 | -3.5360 |
| RP11-515I12.1 | 0.5645 | 0.9878 | 2.7855 | 6.16E-03 | 1.42E-01 | -3.5613 |
| H2BC20P | -0.5341 | 2.1010 | -2.7808 | 6.25E-03 | 1.43E-01 | -3.5737 |
| ITGB2 | 0.8408 | 4.6946 | 2.7802 | 6.26E-03 | 1.43E-01 | -3.5753 |
| CPQ | 0.5819 | 3.1387 | 2.7772 | 6.31E-03 | 1.44E-01 | -3.5830 |
| RP11-147L13.12 | 0.6237 | 3.3085 | 2.7758 | 6.34E-03 | 1.44E-01 | -3.5869 |
| UBE2S | -0.6489 | 2.7659 | -2.7736 | 6.38E-03 | 1.44E-01 | -3.5925 |
| TSPOAP1 | 0.6161 | 1.6690 | 2.7700 | 6.44E-03 | 1.45E-01 | -3.6019 |
| OLFML1 | 0.5103 | 1.7489 | 2.7696 | 6.45E-03 | 1.45E-01 | -3.6030 |
| RNASE1 | 1.1469 | 7.8883 | 2.7692 | 6.46E-03 | 1.45E-01 | -3.6042 |
| KCNJ15 | 1.0713 | 2.2063 | 2.7683 | 6.48E-03 | 1.45E-01 | -3.6066 |
| DDAH1 | 0.5503 | 4.3125 | 2.7681 | 6.48E-03 | 1.45E-01 | -3.6071 |
| HERC5 | 0.5459 | 1.6461 | 2.7658 | 6.52E-03 | 1.45E-01 | -3.6130 |
| CCN1 | 0.9350 | 5.9607 | 2.7637 | 6.56E-03 | 1.46E-01 | -3.6187 |
| SH3BGRL2 | 0.6757 | 3.5250 | 2.7625 | 6.59E-03 | 1.46E-01 | -3.6218 |
| CUL4A | -0.5233 | 3.5779 | -2.7620 | 6.59E-03 | 1.46E-01 | -3.6230 |
| F13A1 | 0.8707 | 2.8004 | 2.7576 | 6.68E-03 | 1.47E-01 | -3.6345 |
| SIGLEC1 | 0.7273 | 2.2604 | 2.7566 | 6.70E-03 | 1.48E-01 | -3.6371 |
| APOBEC3C | 0.6538 | 3.9799 | 2.7564 | 6.70E-03 | 1.48E-01 | -3.6376 |
| DPT | 1.0114 | 3.2925 | 2.7521 | 6.78E-03 | 1.49E-01 | -3.6488 |
| SLC24A3 | 0.6779 | 1.7032 | 2.7510 | 6.81E-03 | 1.49E-01 | -3.6517 |
| SLC11A1 | 0.6712 | 2.3314 | 2.7507 | 6.81E-03 | 1.49E-01 | -3.6525 |
| ANGPT1 | 0.5562 | 1.0935 | 2.7500 | 6.83E-03 | 1.49E-01 | -3.6543 |
| PAPSS2 | 0.7513 | 4.1665 | 2.7496 | 6.83E-03 | 1.49E-01 | -3.6554 |
| H2BC18 | -0.5190 | 0.8118 | -2.7477 | 6.87E-03 | 1.49E-01 | -3.6604 |
| B4GALNT4 | -0.9806 | 1.8039 | -2.7470 | 6.89E-03 | 1.49E-01 | -3.6621 |
| CTD-2591A6.2 | -0.5553 | 0.3486 | -2.7447 | 6.93E-03 | 1.50E-01 | -3.6681 |
| CCL13 | 1.0064 | 3.0851 | 2.7442 | 6.94E-03 | 1.50E-01 | -3.6694 |
| FPR3 | 0.7291 | 3.3215 | 2.7426 | 6.97E-03 | 1.50E-01 | -3.6737 |
| TGM2 | 0.7434 | 5.7183 | 2.7422 | 6.98E-03 | 1.50E-01 | -3.6747 |
| H2AC7 | -0.7477 | 0.9408 | -2.7419 | 6.99E-03 | 1.50E-01 | -3.6755 |
| INPP5J | 0.5077 | 1.3954 | 2.7347 | 7.13E-03 | 1.52E-01 | -3.6941 |
| COL14A1 | 0.8322 | 2.4267 | 2.7315 | 7.20E-03 | 1.52E-01 | -3.7023 |
| SEC14L6 | 0.7649 | 1.5193 | 2.7300 | 7.23E-03 | 1.53E-01 | -3.7063 |
| PLEKHO2 | 0.5216 | 3.6749 | 2.7283 | 7.26E-03 | 1.53E-01 | -3.7106 |
| CDK5R2 | -0.6569 | 0.3983 | -2.7218 | 7.40E-03 | 1.54E-01 | -3.7276 |
| GRAMD2A | 0.7424 | 2.2550 | 2.7177 | 7.49E-03 | 1.55E-01 | -3.7380 |
| TEDC2 | -0.5029 | 1.7757 | -2.7147 | 7.55E-03 | 1.55E-01 | -3.7457 |
| TIMP2 | 0.6554 | 5.5504 | 2.7144 | 7.56E-03 | 1.55E-01 | -3.7465 |
| CYP27A1 | 0.7693 | 4.4022 | 2.7128 | 7.59E-03 | 1.56E-01 | -3.7507 |
| LGALS3 | 0.5410 | 7.0865 | 2.7126 | 7.60E-03 | 1.56E-01 | -3.7512 |
| RAPGEF5 | 0.5071 | 2.4678 | 2.7125 | 7.60E-03 | 1.56E-01 | -3.7513 |
| SCN7A | 0.6454 | 1.2885 | 2.7113 | 7.63E-03 | 1.56E-01 | -3.7546 |
| HMMR | -0.5780 | 1.7584 | -2.7096 | 7.66E-03 | 1.56E-01 | -3.7588 |
| VIM | 0.6123 | 6.9825 | 2.7091 | 7.67E-03 | 1.56E-01 | -3.7601 |
| LRRC31 | 0.9239 | 1.1642 | 2.7090 | 7.68E-03 | 1.56E-01 | -3.7604 |
| RAB3B | -0.5051 | 0.4174 | -2.7087 | 7.68E-03 | 1.56E-01 | -3.7612 |
| EFEMP2 | 0.5836 | 3.1582 | 2.7053 | 7.76E-03 | 1.57E-01 | -3.7699 |
| GPR160 | 0.7484 | 3.0898 | 2.7043 | 7.78E-03 | 1.57E-01 | -3.7726 |
| TBX2 | 0.6331 | 2.4734 | 2.7032 | 7.80E-03 | 1.58E-01 | -3.7753 |
| SPTBN2 | -0.5303 | 2.8376 | -2.6998 | 7.88E-03 | 1.59E-01 | -3.7839 |
| SERPING1 | 0.6545 | 6.2862 | 2.6987 | 7.91E-03 | 1.59E-01 | -3.7869 |
| C16orf89 | 1.6779 | 5.7359 | 2.6973 | 7.94E-03 | 1.59E-01 | -3.7905 |
| NSMF | -0.5304 | 3.6708 | -2.6969 | 7.95E-03 | 1.59E-01 | -3.7915 |
| SLC15A2 | 0.7105 | 2.1088 | 2.6969 | 7.95E-03 | 1.59E-01 | -3.7916 |
| C3AR1 | 0.6836 | 3.1399 | 2.6967 | 7.95E-03 | 1.59E-01 | -3.7920 |
| LRRC75B | 0.5857 | 2.3899 | 2.6963 | 7.96E-03 | 1.59E-01 | -3.7929 |
| RRAS2 | 0.5280 | 3.0212 | 2.6959 | 7.97E-03 | 1.59E-01 | -3.7939 |
| GSTP1 | -0.5810 | 8.3743 | -2.6959 | 7.97E-03 | 1.59E-01 | -3.7941 |
| PPP1R14B | -0.5600 | 5.8060 | -2.6933 | 8.03E-03 | 1.59E-01 | -3.8007 |
| FAM162B | 0.6001 | 1.4709 | 2.6932 | 8.03E-03 | 1.59E-01 | -3.8010 |
| P3H2 | 0.9358 | 2.2032 | 2.6920 | 8.06E-03 | 1.60E-01 | -3.8040 |
| BUB1B | -0.6061 | 1.8245 | -2.6918 | 8.06E-03 | 1.60E-01 | -3.8045 |
| JAML | 0.6101 | 1.9548 | 2.6909 | 8.08E-03 | 1.60E-01 | -3.8068 |
| SFTPB | 1.8929 | 9.9417 | 2.6886 | 8.13E-03 | 1.60E-01 | -3.8126 |
| EVA1A | 0.7535 | 3.1629 | 2.6885 | 8.14E-03 | 1.60E-01 | -3.8129 |
| FUT2 | -0.7131 | 2.8383 | -2.6884 | 8.14E-03 | 1.60E-01 | -3.8131 |
| TFAP2C | 0.6410 | 2.9014 | 2.6877 | 8.16E-03 | 1.61E-01 | -3.8151 |
| TRIM16 | -0.6320 | 2.0624 | -2.6872 | 8.17E-03 | 1.61E-01 | -3.8162 |
| PMP22 | 0.6087 | 4.4274 | 2.6869 | 8.17E-03 | 1.61E-01 | -3.8170 |
| PRSS21 | 1.0076 | 1.2794 | 2.6866 | 8.18E-03 | 1.61E-01 | -3.8177 |
| MSRB3 | 0.5716 | 2.0764 | 2.6853 | 8.21E-03 | 1.61E-01 | -3.8210 |
| MIAT | -0.7437 | 1.5404 | -2.6843 | 8.23E-03 | 1.61E-01 | -3.8235 |
| COL22A1 | -0.5249 | 0.6429 | -2.6842 | 8.24E-03 | 1.61E-01 | -3.8239 |
| CAVIN2 | 0.9262 | 3.2524 | 2.6831 | 8.26E-03 | 1.61E-01 | -3.8268 |
| TCF21 | 0.6686 | 1.1231 | 2.6819 | 8.29E-03 | 1.61E-01 | -3.8297 |
| SPARCL1 | 0.7770 | 5.2343 | 2.6788 | 8.36E-03 | 1.62E-01 | -3.8375 |
| VASN | 0.7130 | 3.9419 | 2.6781 | 8.38E-03 | 1.62E-01 | -3.8393 |
| CCL23 | 0.6044 | 1.0928 | 2.6760 | 8.43E-03 | 1.63E-01 | -3.8447 |
| ACHE | 1.0104 | 2.3539 | 2.6745 | 8.47E-03 | 1.63E-01 | -3.8486 |
| PLCH1 | 0.6184 | 2.2301 | 2.6737 | 8.48E-03 | 1.63E-01 | -3.8507 |
| EZH2 | -0.5263 | 2.5045 | -2.6735 | 8.49E-03 | 1.63E-01 | -3.8512 |
| CTSZ | 0.5019 | 7.4660 | 2.6683 | 8.61E-03 | 1.65E-01 | -3.8642 |
| TCN2 | 0.5989 | 4.1554 | 2.6679 | 8.62E-03 | 1.65E-01 | -3.8654 |
| PLK1 | -0.6414 | 2.3820 | -2.6668 | 8.65E-03 | 1.65E-01 | -3.8681 |
| PTPN13 | 0.9737 | 2.7617 | 2.6663 | 8.66E-03 | 1.65E-01 | -3.8695 |
| CADM1 | 0.9068 | 2.9957 | 2.6653 | 8.69E-03 | 1.65E-01 | -3.8720 |
| CTSH | 1.0047 | 6.0392 | 2.6644 | 8.71E-03 | 1.66E-01 | -3.8742 |
| FGFR3 | 1.0032 | 2.7861 | 2.6642 | 8.71E-03 | 1.66E-01 | -3.8747 |
| F12 | -0.5507 | 1.3947 | -2.6642 | 8.71E-03 | 1.66E-01 | -3.8748 |
| BMP5 | 0.8524 | 1.9089 | 2.6624 | 8.76E-03 | 1.66E-01 | -3.8794 |
| BCAN-AS1 | -0.7634 | 0.8605 | -2.6577 | 8.87E-03 | 1.67E-01 | -3.8912 |
| MIR223HG | 0.5318 | 0.9987 | 2.6554 | 8.93E-03 | 1.68E-01 | -3.8969 |
| FOLR2 | 0.7995 | 3.3872 | 2.6536 | 8.98E-03 | 1.68E-01 | -3.9015 |
| CLEC2B | 0.6093 | 2.4858 | 2.6517 | 9.03E-03 | 1.69E-01 | -3.9064 |
| LGALS2 | 0.7199 | 2.1682 | 2.6511 | 9.04E-03 | 1.69E-01 | -3.9077 |
| WDR91 | 0.5489 | 2.9488 | 2.6509 | 9.04E-03 | 1.69E-01 | -3.9082 |
| CD300LF | 0.6695 | 2.2040 | 2.6471 | 9.14E-03 | 1.70E-01 | -3.9179 |
| SLC2A1 | -1.0201 | 4.3870 | -2.6462 | 9.17E-03 | 1.70E-01 | -3.9202 |
| LINC02315 | -0.6510 | 0.5452 | -2.6434 | 9.24E-03 | 1.71E-01 | -3.9273 |
| CTA-29F11.1 | -0.5036 | 2.3821 | -2.6430 | 9.25E-03 | 1.71E-01 | -3.9283 |
| HLA-DPA1 | 0.9590 | 6.1154 | 2.6417 | 9.28E-03 | 1.71E-01 | -3.9315 |
| HACD1 | -0.5457 | 1.3594 | -2.6397 | 9.33E-03 | 1.72E-01 | -3.9365 |
| TMEM100 | 0.9349 | 1.4796 | 2.6364 | 9.42E-03 | 1.73E-01 | -3.9447 |
| DLX3 | 0.6968 | 1.2218 | 2.6360 | 9.43E-03 | 1.73E-01 | -3.9457 |
| ASPHD1 | -0.9435 | 2.2673 | -2.6346 | 9.47E-03 | 1.73E-01 | -3.9492 |
| TMSB4X | 0.5126 | 9.8615 | 2.6339 | 9.49E-03 | 1.73E-01 | -3.9510 |
| CTD-2245E15.3 | 1.1132 | 2.1287 | 2.6336 | 9.50E-03 | 1.73E-01 | -3.9519 |
| CD44 | 0.6637 | 4.7687 | 2.6318 | 9.54E-03 | 1.73E-01 | -3.9563 |
| SELP | 0.5548 | 1.5386 | 2.6313 | 9.55E-03 | 1.74E-01 | -3.9575 |
| DLC1 | 0.7870 | 2.7784 | 2.6292 | 9.61E-03 | 1.74E-01 | -3.9629 |
| TRIM22 | 0.6629 | 3.7774 | 2.6291 | 9.61E-03 | 1.74E-01 | -3.9630 |
| CRYAB | 0.6312 | 1.8328 | 2.6269 | 9.67E-03 | 1.75E-01 | -3.9686 |
| HSPB8 | 0.7415 | 3.2113 | 2.6264 | 9.69E-03 | 1.75E-01 | -3.9697 |
| LAIR1 | 0.5873 | 2.4107 | 2.6236 | 9.76E-03 | 1.76E-01 | -3.9767 |
| GAPDHP35 | -0.6469 | 0.8790 | -2.6206 | 9.84E-03 | 1.77E-01 | -3.9842 |
| MS4A6A | 0.6611 | 3.0024 | 2.6196 | 9.87E-03 | 1.77E-01 | -3.9867 |
| SDC3 | 0.5703 | 4.0944 | 2.6185 | 9.90E-03 | 1.77E-01 | -3.9895 |
| LCP2 | 0.5363 | 2.5418 | 2.6179 | 9.92E-03 | 1.77E-01 | -3.9910 |
| CXCL17 | 1.2371 | 7.2724 | 2.6172 | 9.94E-03 | 1.78E-01 | -3.9927 |
| HPN | 0.8431 | 4.1923 | 2.6165 | 9.96E-03 | 1.78E-01 | -3.9946 |
| MAOB | 0.7417 | 2.4861 | 2.6163 | 9.96E-03 | 1.78E-01 | -3.9949 |
| LINC02889 | 1.1887 | 2.9943 | 2.6151 | 9.99E-03 | 1.78E-01 | -3.9980 |
| NFIX | 0.8045 | 3.5385 | 2.6145 | 1.00E-02 | 1.78E-01 | -3.9995 |
| RETN | 1.0047 | 1.7102 | 2.6122 | 1.01E-02 | 1.79E-01 | -4.0052 |
| GAS5 | -0.5862 | 5.5951 | -2.6113 | 1.01E-02 | 1.79E-01 | -4.0075 |
| CTSV | -0.7075 | 1.1806 | -2.6107 | 1.01E-02 | 1.79E-01 | -4.0091 |
| NKD2 | 0.6379 | 2.2614 | 2.6054 | 1.03E-02 | 1.80E-01 | -4.0221 |
| HBB | 1.3006 | 3.5019 | 2.6052 | 1.03E-02 | 1.80E-01 | -4.0227 |
| MXRA8 | 0.7306 | 4.6849 | 2.6028 | 1.03E-02 | 1.81E-01 | -4.0287 |
| CMAHP | 0.7498 | 2.9571 | 2.6015 | 1.04E-02 | 1.81E-01 | -4.0318 |
| TMPRSS4 | 0.9509 | 3.0621 | 2.5995 | 1.04E-02 | 1.81E-01 | -4.0367 |
| PSPHP1 | -1.8957 | 2.2953 | -2.5978 | 1.05E-02 | 1.82E-01 | -4.0411 |
| FGR | 0.6402 | 2.7957 | 2.5961 | 1.05E-02 | 1.82E-01 | -4.0452 |
| H2BC21 | -0.8598 | 3.8227 | -2.5960 | 1.05E-02 | 1.82E-01 | -4.0454 |
| BCL2A1 | 0.6209 | 2.9138 | 2.5955 | 1.06E-02 | 1.82E-01 | -4.0467 |
| NEK2 | -0.6814 | 2.0960 | -2.5949 | 1.06E-02 | 1.82E-01 | -4.0483 |
| ADH1B | 1.1134 | 2.1902 | 2.5942 | 1.06E-02 | 1.82E-01 | -4.0498 |
| EDNRA | 0.6267 | 2.5873 | 2.5940 | 1.06E-02 | 1.82E-01 | -4.0504 |
| CMAS | -0.5015 | 4.2234 | -2.5904 | 1.07E-02 | 1.83E-01 | -4.0593 |
| MFAP2 | 0.7132 | 3.0538 | 2.5903 | 1.07E-02 | 1.83E-01 | -4.0596 |
| COL16A1 | 0.5849 | 2.9011 | 2.5898 | 1.07E-02 | 1.83E-01 | -4.0607 |
| CD14 | 0.6359 | 5.3659 | 2.5892 | 1.07E-02 | 1.84E-01 | -4.0623 |
| EGFR | 0.7991 | 3.4850 | 2.5868 | 1.08E-02 | 1.84E-01 | -4.0682 |
| TFDP1 | -0.5475 | 4.2859 | -2.5836 | 1.09E-02 | 1.86E-01 | -4.0761 |
| LRIG3 | 0.7479 | 3.1937 | 2.5831 | 1.09E-02 | 1.86E-01 | -4.0774 |
| MTUS1 | 0.6101 | 3.7969 | 2.5825 | 1.09E-02 | 1.86E-01 | -4.0788 |
| WDFY4 | 0.5292 | 1.5091 | 2.5823 | 1.09E-02 | 1.86E-01 | -4.0793 |
| DKK3 | 0.7225 | 3.6557 | 2.5816 | 1.10E-02 | 1.86E-01 | -4.0810 |
| H2BC5 | -0.8590 | 4.3920 | -2.5811 | 1.10E-02 | 1.86E-01 | -4.0821 |
| PPARGC1A | -0.5452 | 0.6703 | -2.5810 | 1.10E-02 | 1.86E-01 | -4.0824 |
| MAML2 | 0.5170 | 2.1650 | 2.5782 | 1.11E-02 | 1.87E-01 | -4.0894 |
| FHL1 | 0.8705 | 2.3193 | 2.5781 | 1.11E-02 | 1.87E-01 | -4.0896 |
| PKP2 | -0.7754 | 1.2704 | -2.5765 | 1.11E-02 | 1.87E-01 | -4.0934 |
| INSM1 | -0.7789 | 0.3125 | -2.5754 | 1.12E-02 | 1.87E-01 | -4.0961 |
| WLS | 0.7661 | 4.0242 | 2.5749 | 1.12E-02 | 1.87E-01 | -4.0974 |
| BCAM | 0.7602 | 6.0060 | 2.5744 | 1.12E-02 | 1.87E-01 | -4.0986 |
| CDH16 | -0.5587 | 0.2924 | -2.5701 | 1.13E-02 | 1.89E-01 | -4.1092 |
| PPP1R14C | 1.0077 | 2.7765 | 2.5695 | 1.13E-02 | 1.89E-01 | -4.1107 |
| CYBRD1 | 0.7343 | 4.2269 | 2.5664 | 1.14E-02 | 1.90E-01 | -4.1184 |
| PGD | -0.7333 | 6.1876 | -2.5641 | 1.15E-02 | 1.90E-01 | -4.1240 |
| LINC02253 | -0.5675 | 0.3602 | -2.5635 | 1.15E-02 | 1.91E-01 | -4.1255 |
| SCTR | 1.1072 | 2.4917 | 2.5634 | 1.15E-02 | 1.91E-01 | -4.1257 |
| ENC1 | 0.7322 | 3.9873 | 2.5591 | 1.17E-02 | 1.92E-01 | -4.1361 |
| HAGLR | 0.9871 | 3.1642 | 2.5577 | 1.17E-02 | 1.92E-01 | -4.1396 |
| NAPSB | 0.7071 | 3.0676 | 2.5563 | 1.18E-02 | 1.93E-01 | -4.1430 |
| IFI44L | 0.8464 | 2.0279 | 2.5539 | 1.18E-02 | 1.94E-01 | -4.1488 |
| LINC01671 | 0.7183 | 1.4205 | 2.5511 | 1.19E-02 | 1.94E-01 | -4.1558 |
| PLEK | 0.7016 | 3.3562 | 2.5503 | 1.19E-02 | 1.95E-01 | -4.1576 |
| SERPIND1 | 1.2698 | 1.7455 | 2.5501 | 1.20E-02 | 1.95E-01 | -4.1581 |
| GGTLC1 | 1.2890 | 2.7548 | 2.5495 | 1.20E-02 | 1.95E-01 | -4.1597 |
| TREM1 | 1.0126 | 3.2684 | 2.5475 | 1.20E-02 | 1.96E-01 | -4.1643 |
| RGL1 | 0.5435 | 3.0768 | 2.5461 | 1.21E-02 | 1.96E-01 | -4.1679 |
| TMEM47 | 0.5483 | 2.1860 | 2.5450 | 1.21E-02 | 1.96E-01 | -4.1705 |
| GAL | -0.7896 | 0.7109 | -2.5450 | 1.21E-02 | 1.96E-01 | -4.1706 |
| CSF3R | 0.8753 | 2.7053 | 2.5435 | 1.22E-02 | 1.97E-01 | -4.1741 |
| PRTFDC1 | -0.5220 | 2.1685 | -2.5434 | 1.22E-02 | 1.97E-01 | -4.1744 |
| LOXL1 | 0.5821 | 3.0403 | 2.5419 | 1.22E-02 | 1.97E-01 | -4.1780 |
| SPN | 0.5964 | 2.0303 | 2.5389 | 1.23E-02 | 1.98E-01 | -4.1853 |
| SRPX | 0.5511 | 1.9775 | 2.5380 | 1.24E-02 | 1.98E-01 | -4.1875 |
| OR7E47P | 0.5030 | 1.3395 | 2.5374 | 1.24E-02 | 1.98E-01 | -4.1890 |
| HERC6 | 0.5397 | 2.3657 | 2.5341 | 1.25E-02 | 1.99E-01 | -4.1969 |
| SERTAD4-AS1 | 0.6047 | 1.9161 | 2.5331 | 1.25E-02 | 1.99E-01 | -4.1994 |
| DPYD | 0.6876 | 3.0045 | 2.5328 | 1.25E-02 | 1.99E-01 | -4.2002 |
| KDR | 0.7386 | 3.1419 | 2.5325 | 1.25E-02 | 1.99E-01 | -4.2007 |
| RP11-598F7.3 | 0.5292 | 0.8619 | 2.5305 | 1.26E-02 | 2.00E-01 | -4.2058 |
| GJA1 | 0.8996 | 4.5310 | 2.5300 | 1.26E-02 | 2.00E-01 | -4.2068 |
| MFSD4A | 1.1084 | 2.7685 | 2.5299 | 1.26E-02 | 2.00E-01 | -4.2072 |
| RNASE2 | 0.5146 | 1.2442 | 2.5240 | 1.28E-02 | 2.02E-01 | -4.2212 |
| PDGFRA | 0.5148 | 2.0616 | 2.5215 | 1.29E-02 | 2.03E-01 | -4.2274 |
| SORL1 | 0.6483 | 2.7260 | 2.5211 | 1.29E-02 | 2.03E-01 | -4.2284 |
| RP11-206M11.7 | -0.5869 | 0.1537 | -2.5201 | 1.30E-02 | 2.03E-01 | -4.2307 |
| RCAN2 | 0.7635 | 2.4548 | 2.5162 | 1.31E-02 | 2.04E-01 | -4.2401 |
| PAQR4 | -0.5077 | 2.6998 | -2.5155 | 1.31E-02 | 2.04E-01 | -4.2419 |
| RNU7-45P | 0.5255 | 1.2378 | 2.5142 | 1.32E-02 | 2.04E-01 | -4.2450 |
| LTK | 0.8907 | 1.9363 | 2.5108 | 1.33E-02 | 2.05E-01 | -4.2530 |
| SULT1A2 | 0.5334 | 1.6117 | 2.5108 | 1.33E-02 | 2.05E-01 | -4.2531 |
| ACVRL1 | 0.5176 | 2.8287 | 2.5091 | 1.34E-02 | 2.06E-01 | -4.2571 |
| TLR4 | 0.5051 | 1.8065 | 2.5083 | 1.34E-02 | 2.06E-01 | -4.2591 |
| SLIT2 | 0.5421 | 1.5632 | 2.5075 | 1.34E-02 | 2.06E-01 | -4.2610 |
| LYZ | 1.1015 | 7.2638 | 2.5070 | 1.34E-02 | 2.06E-01 | -4.2621 |
| CD1B | 0.5010 | 0.7772 | 2.5030 | 1.36E-02 | 2.07E-01 | -4.2717 |
| SIX1 | 0.8265 | 2.3504 | 2.5012 | 1.36E-02 | 2.08E-01 | -4.2760 |
| RASSF2 | 0.5451 | 2.7171 | 2.4983 | 1.38E-02 | 2.10E-01 | -4.2831 |
| PECAM1 | 0.5224 | 4.4688 | 2.4963 | 1.38E-02 | 2.10E-01 | -4.2880 |
| HLA-DRB6 | 1.1666 | 4.8632 | 2.4942 | 1.39E-02 | 2.11E-01 | -4.2928 |
| TGFB1 | 0.5021 | 4.6237 | 2.4934 | 1.39E-02 | 2.11E-01 | -4.2947 |
| COLEC12 | 0.7192 | 2.3605 | 2.4925 | 1.40E-02 | 2.11E-01 | -4.2968 |
| MEGF6 | 0.7099 | 3.1195 | 2.4922 | 1.40E-02 | 2.11E-01 | -4.2977 |
| EVI2A | 0.5108 | 2.4730 | 2.4908 | 1.40E-02 | 2.12E-01 | -4.3009 |
| SGPP2 | 0.7627 | 4.2068 | 2.4904 | 1.40E-02 | 2.12E-01 | -4.3020 |
| RP11-295G20.2 | -0.7928 | 2.5160 | -2.4897 | 1.41E-02 | 2.12E-01 | -4.3036 |
| GABRE | 0.7306 | 1.8942 | 2.4881 | 1.41E-02 | 2.12E-01 | -4.3073 |
| LYPD3 | -1.0188 | 2.2549 | -2.4870 | 1.42E-02 | 2.13E-01 | -4.3101 |
| CFI | 0.7848 | 4.2130 | 2.4869 | 1.42E-02 | 2.13E-01 | -4.3103 |
| METTL7A | 0.6876 | 3.9913 | 2.4834 | 1.43E-02 | 2.14E-01 | -4.3187 |
| FOXF2 | 0.6326 | 1.9216 | 2.4811 | 1.44E-02 | 2.15E-01 | -4.3240 |
| H2AC6 | -0.8385 | 4.8426 | -2.4804 | 1.44E-02 | 2.15E-01 | -4.3257 |
| HLA-DQB1 | 0.9828 | 5.8343 | 2.4796 | 1.45E-02 | 2.15E-01 | -4.3276 |
| AQP4 | 1.2654 | 2.8811 | 2.4779 | 1.45E-02 | 2.15E-01 | -4.3316 |
| CSF2RB | 0.5749 | 2.5018 | 2.4756 | 1.46E-02 | 2.16E-01 | -4.3371 |
| COL27A1 | 0.5212 | 1.8518 | 2.4736 | 1.47E-02 | 2.16E-01 | -4.3419 |
| PPP1R14B-AS1 | -0.5374 | 1.6658 | -2.4734 | 1.47E-02 | 2.16E-01 | -4.3423 |
| LPCAT1 | 0.8228 | 6.6557 | 2.4715 | 1.48E-02 | 2.17E-01 | -4.3469 |
| PLAAT3 | 0.8067 | 4.1342 | 2.4699 | 1.48E-02 | 2.18E-01 | -4.3505 |
| ARHGAP27P1-BPTFP1-KPNA2P3 | 0.5261 | 2.1673 | 2.4692 | 1.49E-02 | 2.18E-01 | -4.3522 |
| ATP2B4 | -0.5473 | 4.1960 | -2.4671 | 1.49E-02 | 2.19E-01 | -4.3572 |
| INHA | -0.9915 | 1.1041 | -2.4671 | 1.49E-02 | 2.19E-01 | -4.3573 |
| FAM83A-AS1 | -0.7827 | 1.6135 | -2.4657 | 1.50E-02 | 2.19E-01 | -4.3606 |
| ITM2A | 0.6856 | 2.9741 | 2.4655 | 1.50E-02 | 2.19E-01 | -4.3611 |
| THBS1 | 0.8000 | 4.7093 | 2.4631 | 1.51E-02 | 2.20E-01 | -4.3668 |
| JCHAIN | 1.0963 | 7.3797 | 2.4620 | 1.52E-02 | 2.20E-01 | -4.3693 |
| CHCHD2P9 | -0.6716 | 2.6505 | -2.4614 | 1.52E-02 | 2.20E-01 | -4.3707 |
| ADAMTSL2 | 0.5456 | 2.3562 | 2.4613 | 1.52E-02 | 2.20E-01 | -4.3709 |
| KCTD3 | -0.5186 | 3.2310 | -2.4596 | 1.52E-02 | 2.21E-01 | -4.3750 |
| VGF | -0.7427 | 0.6277 | -2.4591 | 1.53E-02 | 2.21E-01 | -4.3761 |
| IL7R | 0.7639 | 2.9269 | 2.4589 | 1.53E-02 | 2.21E-01 | -4.3765 |
| HIGD1B | 0.6231 | 1.8464 | 2.4588 | 1.53E-02 | 2.21E-01 | -4.3769 |
| NIBAN1 | 0.7033 | 3.1193 | 2.4587 | 1.53E-02 | 2.21E-01 | -4.3771 |
| RPL41P5 | -0.5807 | 5.5620 | -2.4556 | 1.54E-02 | 2.22E-01 | -4.3843 |
| PLAAT4 | 0.9403 | 5.5694 | 2.4550 | 1.54E-02 | 2.22E-01 | -4.3859 |
| SFRP4 | 0.9535 | 3.5893 | 2.4522 | 1.56E-02 | 2.23E-01 | -4.3924 |
| LAPTM5 | 0.6325 | 6.2289 | 2.4493 | 1.57E-02 | 2.24E-01 | -4.3992 |
| LTBP2 | 0.7178 | 4.4466 | 2.4480 | 1.57E-02 | 2.24E-01 | -4.4022 |
| EID3 | -0.5503 | 1.1994 | -2.4476 | 1.57E-02 | 2.25E-01 | -4.4032 |
| SLC4A4 | 0.7052 | 2.1115 | 2.4467 | 1.58E-02 | 2.25E-01 | -4.4053 |
| LGALS9 | 0.6275 | 3.9961 | 2.4456 | 1.58E-02 | 2.25E-01 | -4.4079 |
| PPP2R2C | -0.7146 | 0.7158 | -2.4449 | 1.59E-02 | 2.25E-01 | -4.4095 |
| MIF | -0.5848 | 5.3089 | -2.4449 | 1.59E-02 | 2.25E-01 | -4.4095 |
| NFE2L3 | 0.6006 | 3.1634 | 2.4449 | 1.59E-02 | 2.25E-01 | -4.4096 |
| SNX10 | 0.5932 | 2.9638 | 2.4401 | 1.61E-02 | 2.27E-01 | -4.4207 |
| EVI2B | 0.6320 | 3.4803 | 2.4398 | 1.61E-02 | 2.27E-01 | -4.4214 |
| KITLG | 0.5898 | 3.6786 | 2.4395 | 1.61E-02 | 2.27E-01 | -4.4221 |
| STAB1 | 0.5084 | 3.0123 | 2.4386 | 1.61E-02 | 2.28E-01 | -4.4241 |
| RAMP2 | 0.6055 | 3.4956 | 2.4370 | 1.62E-02 | 2.28E-01 | -4.4280 |
| WNT2 | 0.5388 | 1.6932 | 2.4364 | 1.62E-02 | 2.28E-01 | -4.4294 |
| MTCO1P53 | -0.5714 | 0.5737 | -2.4361 | 1.62E-02 | 2.28E-01 | -4.4300 |
| SFTPD | 1.5150 | 6.1405 | 2.4359 | 1.62E-02 | 2.28E-01 | -4.4305 |
| LRRK2-DT | 0.8364 | 1.5657 | 2.4333 | 1.63E-02 | 2.30E-01 | -4.4366 |
| RSAD2 | 0.6588 | 2.1636 | 2.4326 | 1.64E-02 | 2.30E-01 | -4.4381 |
| SEMA5A | 0.5574 | 1.2989 | 2.4326 | 1.64E-02 | 2.30E-01 | -4.4382 |
| FCER1G | 0.6469 | 5.4244 | 2.4317 | 1.64E-02 | 2.30E-01 | -4.4403 |
| AC008268.1 | 1.2328 | 1.6979 | 2.4314 | 1.64E-02 | 2.30E-01 | -4.4410 |
| COMP | 1.1591 | 3.2692 | 2.4309 | 1.65E-02 | 2.30E-01 | -4.4422 |
| CSTA | 0.6731 | 2.3339 | 2.4306 | 1.65E-02 | 2.30E-01 | -4.4429 |
| LSP1 | 0.5666 | 3.8140 | 2.4302 | 1.65E-02 | 2.30E-01 | -4.4438 |
| ROS1 | 1.0362 | 3.0600 | 2.4298 | 1.65E-02 | 2.30E-01 | -4.4447 |
| EMCN | 0.5395 | 1.5964 | 2.4297 | 1.65E-02 | 2.30E-01 | -4.4450 |
| HBA2 | 1.0488 | 2.4550 | 2.4280 | 1.66E-02 | 2.30E-01 | -4.4490 |
| HLA-DMB | 0.7113 | 4.1941 | 2.4280 | 1.66E-02 | 2.30E-01 | -4.4490 |
| PALM3 | 0.8976 | 2.3244 | 2.4279 | 1.66E-02 | 2.30E-01 | -4.4491 |
| PDPN | 0.6460 | 2.7174 | 2.4275 | 1.66E-02 | 2.30E-01 | -4.4500 |
| CENPU | -0.5419 | 2.3835 | -2.4273 | 1.66E-02 | 2.30E-01 | -4.4505 |
| KRT15 | 1.0513 | 2.5254 | 2.4263 | 1.67E-02 | 2.31E-01 | -4.4528 |
| SLC16A14 | -0.9106 | 2.1183 | -2.4263 | 1.67E-02 | 2.31E-01 | -4.4529 |
| PEBP4 | 1.2462 | 2.8040 | 2.4248 | 1.67E-02 | 2.31E-01 | -4.4563 |
| RP11-190J1.3 | -0.5216 | 0.5715 | -2.4237 | 1.68E-02 | 2.32E-01 | -4.4589 |
| ARSD | 0.5439 | 4.0987 | 2.4236 | 1.68E-02 | 2.32E-01 | -4.4592 |
| ANXA3 | 0.6548 | 3.0809 | 2.4220 | 1.68E-02 | 2.32E-01 | -4.4628 |
| SLC27A2 | -0.6634 | 2.1477 | -2.4207 | 1.69E-02 | 2.32E-01 | -4.4659 |
| LRRN4 | 1.0816 | 2.5424 | 2.4165 | 1.71E-02 | 2.34E-01 | -4.4756 |
| CEACAM4 | 0.5096 | 1.0257 | 2.4150 | 1.72E-02 | 2.35E-01 | -4.4791 |
| MICAL2 | 0.5282 | 3.1119 | 2.4148 | 1.72E-02 | 2.35E-01 | -4.4797 |
| HLA-H | 0.6708 | 5.3914 | 2.4143 | 1.72E-02 | 2.35E-01 | -4.4808 |
| RP1-27K12.4 | -0.5096 | 0.6649 | -2.4136 | 1.72E-02 | 2.35E-01 | -4.4824 |
| H2AC13 | -0.5802 | 1.0339 | -2.4134 | 1.72E-02 | 2.35E-01 | -4.4829 |
| ITGBL1 | 0.5755 | 1.8944 | 2.4125 | 1.73E-02 | 2.35E-01 | -4.4850 |
| PTPRU | 0.7027 | 3.6382 | 2.4123 | 1.73E-02 | 2.35E-01 | -4.4854 |
| SLC22A31 | 1.1913 | 5.3410 | 2.4118 | 1.73E-02 | 2.36E-01 | -4.4865 |
| MATN3 | 0.5480 | 1.6389 | 2.4101 | 1.74E-02 | 2.36E-01 | -4.4906 |
| PBK | -0.6317 | 1.8815 | -2.4086 | 1.74E-02 | 2.36E-01 | -4.4940 |
| FOXA2 | 1.0272 | 3.1093 | 2.4061 | 1.76E-02 | 2.37E-01 | -4.4996 |
| GGT1 | 0.6195 | 2.9350 | 2.4060 | 1.76E-02 | 2.37E-01 | -4.5000 |
| SERTAD4 | 0.6085 | 1.9578 | 2.4057 | 1.76E-02 | 2.37E-01 | -4.5007 |
| SLCO2B1 | 0.6420 | 3.0319 | 2.4053 | 1.76E-02 | 2.37E-01 | -4.5017 |
| AQP9 | 0.5759 | 1.7845 | 2.4052 | 1.76E-02 | 2.37E-01 | -4.5018 |
| TSKU | -0.7211 | 3.8740 | -2.4046 | 1.76E-02 | 2.38E-01 | -4.5032 |
| ZFP42 | -0.6402 | 0.4306 | -2.4026 | 1.77E-02 | 2.38E-01 | -4.5078 |
| NFAM1 | 0.5112 | 1.9235 | 2.4015 | 1.78E-02 | 2.38E-01 | -4.5103 |
| EDIL3 | 0.7049 | 2.6309 | 2.4004 | 1.78E-02 | 2.38E-01 | -4.5130 |
| NPR1 | 0.5742 | 1.7310 | 2.3976 | 1.80E-02 | 2.40E-01 | -4.5194 |
| ACP5 | 0.5832 | 5.2758 | 2.3965 | 1.80E-02 | 2.40E-01 | -4.5218 |
| HLA-DRB1 | 0.8817 | 9.0600 | 2.3904 | 1.83E-02 | 2.42E-01 | -4.5358 |
| GPR68 | 0.5994 | 2.4393 | 2.3903 | 1.83E-02 | 2.42E-01 | -4.5362 |
| GNG11 | 0.6481 | 3.1813 | 2.3902 | 1.83E-02 | 2.42E-01 | -4.5363 |
| RP11-357D18.1 | 0.6049 | 0.8266 | 2.3873 | 1.84E-02 | 2.43E-01 | -4.5431 |
| VCAM1 | 0.6263 | 2.4699 | 2.3870 | 1.85E-02 | 2.43E-01 | -4.5438 |
| IRS2 | -0.7361 | 2.5531 | -2.3862 | 1.85E-02 | 2.43E-01 | -4.5456 |
| HAS3 | 0.9773 | 2.8768 | 2.3858 | 1.85E-02 | 2.43E-01 | -4.5464 |
| GMFG | 0.5493 | 3.8016 | 2.3842 | 1.86E-02 | 2.44E-01 | -4.5500 |
| TNFRSF19 | 0.6546 | 2.5008 | 2.3829 | 1.87E-02 | 2.45E-01 | -4.5531 |
| AC005336.4 | -0.6989 | 0.6514 | -2.3826 | 1.87E-02 | 2.45E-01 | -4.5537 |
| PRSS12 | 0.7503 | 1.3977 | 2.3817 | 1.87E-02 | 2.45E-01 | -4.5559 |
| HLA-DRA | 0.8214 | 9.7629 | 2.3814 | 1.87E-02 | 2.45E-01 | -4.5565 |
| LDLRAD1 | -0.8382 | 1.4731 | -2.3765 | 1.90E-02 | 2.47E-01 | -4.5676 |
| CTSS | 0.5752 | 5.7399 | 2.3726 | 1.92E-02 | 2.48E-01 | -4.5766 |
| CENPF | -0.5456 | 2.1150 | -2.3723 | 1.92E-02 | 2.49E-01 | -4.5773 |
| FLRT3 | 0.7630 | 2.7741 | 2.3718 | 1.92E-02 | 2.49E-01 | -4.5784 |
| SIK1 | -0.6427 | 0.8673 | -2.3703 | 1.93E-02 | 2.49E-01 | -4.5819 |
| LRP1 | 0.5007 | 4.2004 | 2.3688 | 1.93E-02 | 2.50E-01 | -4.5853 |
| KLRG2 | 0.5657 | 0.9938 | 2.3686 | 1.94E-02 | 2.50E-01 | -4.5857 |
| POPDC3 | -0.6149 | 0.5670 | -2.3645 | 1.96E-02 | 2.51E-01 | -4.5950 |
| CYP1B1 | 0.6740 | 3.2477 | 2.3632 | 1.96E-02 | 2.52E-01 | -4.5981 |
| ESAM | 0.6446 | 3.9719 | 2.3612 | 1.97E-02 | 2.52E-01 | -4.6026 |
| H3C4 | -0.7206 | 1.3508 | -2.3598 | 1.98E-02 | 2.53E-01 | -4.6058 |
| LACTB2 | 0.5157 | 4.0323 | 2.3588 | 1.99E-02 | 2.53E-01 | -4.6080 |
| RP11-93B14.9 | 0.5562 | 2.0438 | 2.3586 | 1.99E-02 | 2.53E-01 | -4.6085 |
| RP11-344B5.2 | 0.5576 | 1.7093 | 2.3580 | 1.99E-02 | 2.53E-01 | -4.6098 |
| STEAP4 | 0.8822 | 3.9246 | 2.3568 | 2.00E-02 | 2.54E-01 | -4.6126 |
| TAFA5 | 0.5193 | 1.4334 | 2.3561 | 2.00E-02 | 2.54E-01 | -4.6140 |
| ATP8B1 | -0.5298 | 3.3277 | -2.3557 | 2.00E-02 | 2.54E-01 | -4.6149 |
| GPRIN1 | -0.5062 | 1.6408 | -2.3551 | 2.00E-02 | 2.54E-01 | -4.6164 |
| SYNGR3 | -0.5789 | 1.0141 | -2.3542 | 2.01E-02 | 2.55E-01 | -4.6184 |
| APLP1 | -0.7781 | 1.7316 | -2.3508 | 2.03E-02 | 2.56E-01 | -4.6262 |
| DAPK1 | 0.5961 | 3.5439 | 2.3498 | 2.03E-02 | 2.56E-01 | -4.6284 |
| PFKP | -0.7787 | 4.8810 | -2.3492 | 2.03E-02 | 2.56E-01 | -4.6297 |
| PER3 | 0.5248 | 2.0848 | 2.3453 | 2.06E-02 | 2.57E-01 | -4.6386 |
| VPS9D1-AS1 | -0.5031 | 2.0215 | -2.3428 | 2.07E-02 | 2.58E-01 | -4.6443 |
| RRM2 | -0.7402 | 3.0050 | -2.3416 | 2.07E-02 | 2.59E-01 | -4.6468 |
| BIN2 | 0.5130 | 2.2035 | 2.3416 | 2.08E-02 | 2.59E-01 | -4.6469 |
| GPRIN2 | 0.7137 | 2.3039 | 2.3352 | 2.11E-02 | 2.61E-01 | -4.6613 |
| CDKL2 | 0.6692 | 1.7868 | 2.3344 | 2.11E-02 | 2.61E-01 | -4.6630 |
| MSN | 0.5012 | 6.5991 | 2.3329 | 2.12E-02 | 2.62E-01 | -4.6666 |
| TUBB3 | -0.5831 | 1.4482 | -2.3289 | 2.14E-02 | 2.64E-01 | -4.6755 |
| RPL9P9 | 0.8208 | 6.4418 | 2.3277 | 2.15E-02 | 2.64E-01 | -4.6782 |
| AQP3 | 1.1148 | 6.6624 | 2.3263 | 2.16E-02 | 2.64E-01 | -4.6812 |
| RP11-1C8.7 | -0.5279 | 0.4544 | -2.3208 | 2.19E-02 | 2.67E-01 | -4.6936 |
| ARHGAP31 | 0.5084 | 2.1931 | 2.3207 | 2.19E-02 | 2.67E-01 | -4.6937 |
| FAM184A | 0.5023 | 1.3825 | 2.3171 | 2.21E-02 | 2.67E-01 | -4.7017 |
| PIK3AP1 | 0.5085 | 2.3447 | 2.3165 | 2.21E-02 | 2.68E-01 | -4.7032 |
| RP11-284F21.10 | -0.9093 | 1.5758 | -2.3157 | 2.22E-02 | 2.68E-01 | -4.7049 |
| ASPN | 0.7727 | 3.1378 | 2.3138 | 2.23E-02 | 2.69E-01 | -4.7092 |
| NEFL | -0.5160 | 0.2979 | -2.3135 | 2.23E-02 | 2.69E-01 | -4.7099 |
| IL17RB | 0.5362 | 2.0208 | 2.3124 | 2.24E-02 | 2.69E-01 | -4.7123 |
| MRC2 | 0.5602 | 4.2699 | 2.3081 | 2.26E-02 | 2.70E-01 | -4.7218 |
| CPLX1 | -0.5375 | 1.6416 | -2.3057 | 2.27E-02 | 2.71E-01 | -4.7272 |
| FIBIN | 0.6092 | 2.0159 | 2.3055 | 2.28E-02 | 2.71E-01 | -4.7276 |
| GNG4 | -0.7142 | 0.7891 | -2.3048 | 2.28E-02 | 2.71E-01 | -4.7292 |
| LRRK2 | 0.9003 | 2.4772 | 2.3031 | 2.29E-02 | 2.72E-01 | -4.7329 |
| TRIM16L | -0.7309 | 1.7362 | -2.3026 | 2.29E-02 | 2.72E-01 | -4.7342 |
| ACSL4 | 0.5113 | 3.5947 | 2.3015 | 2.30E-02 | 2.72E-01 | -4.7364 |
| MIR186 | 0.5611 | 2.4269 | 2.3006 | 2.30E-02 | 2.72E-01 | -4.7384 |
| PTCSC3 | 0.7080 | 1.3653 | 2.3003 | 2.31E-02 | 2.72E-01 | -4.7391 |
| HLA-J | 0.6951 | 2.0929 | 2.2974 | 2.32E-02 | 2.73E-01 | -4.7457 |
| ANGPTL2 | 0.6035 | 3.6497 | 2.2961 | 2.33E-02 | 2.74E-01 | -4.7484 |
| MX1 | 0.7862 | 3.7199 | 2.2957 | 2.33E-02 | 2.74E-01 | -4.7493 |
| SPOCK2 | 0.7238 | 3.4508 | 2.2940 | 2.34E-02 | 2.74E-01 | -4.7531 |
| HHIPL2 | -0.8676 | 1.3170 | -2.2925 | 2.35E-02 | 2.75E-01 | -4.7564 |
| TMEM178A | 0.5696 | 1.2226 | 2.2904 | 2.36E-02 | 2.76E-01 | -4.7612 |
| RGS10 | 0.5447 | 4.3574 | 2.2897 | 2.37E-02 | 2.76E-01 | -4.7625 |
| RP1-34B20.4 | -0.5600 | 0.7906 | -2.2852 | 2.40E-02 | 2.78E-01 | -4.7726 |
| HHIP | 0.6780 | 0.8673 | 2.2835 | 2.41E-02 | 2.78E-01 | -4.7764 |
| CCL21 | 0.8617 | 5.0913 | 2.2832 | 2.41E-02 | 2.78E-01 | -4.7770 |
| IFIT3 | 0.6294 | 3.8619 | 2.2825 | 2.41E-02 | 2.79E-01 | -4.7784 |
| SIRPA | 0.5338 | 3.3840 | 2.2776 | 2.44E-02 | 2.81E-01 | -4.7893 |
| HGD | -1.0879 | 2.2503 | -2.2769 | 2.45E-02 | 2.81E-01 | -4.7908 |
| PGM5 | 0.5030 | 1.1244 | 2.2753 | 2.46E-02 | 2.82E-01 | -4.7942 |
| CD74 | 0.7526 | 9.9665 | 2.2752 | 2.46E-02 | 2.82E-01 | -4.7946 |
| TNXB | 0.5652 | 1.2742 | 2.2750 | 2.46E-02 | 2.82E-01 | -4.7949 |
| SAPCD2 | -0.5906 | 2.2088 | -2.2747 | 2.46E-02 | 2.82E-01 | -4.7957 |
| ACAD8 | 0.6019 | 3.2653 | 2.2746 | 2.46E-02 | 2.82E-01 | -4.7959 |
| POF1B | -0.7040 | 1.5412 | -2.2729 | 2.47E-02 | 2.82E-01 | -4.7996 |
| AEBP1 | 0.6874 | 6.0134 | 2.2727 | 2.47E-02 | 2.82E-01 | -4.8000 |
| SYT13 | -0.9239 | 0.8975 | -2.2727 | 2.47E-02 | 2.82E-01 | -4.8000 |
| SPRR3 | -0.6939 | 0.4945 | -2.2727 | 2.47E-02 | 2.82E-01 | -4.8000 |
| CTSF | 0.5732 | 4.0279 | 2.2722 | 2.47E-02 | 2.82E-01 | -4.8011 |
| FILIP1 | 0.5211 | 1.2440 | 2.2712 | 2.48E-02 | 2.83E-01 | -4.8034 |
| CYP4F3 | -0.8593 | 0.7849 | -2.2696 | 2.49E-02 | 2.83E-01 | -4.8068 |
| MIR6772 | 0.5579 | 2.5439 | 2.2694 | 2.49E-02 | 2.83E-01 | -4.8073 |
| CALCA | -1.8340 | 1.2875 | -2.2693 | 2.49E-02 | 2.83E-01 | -4.8076 |
| MAFK | -0.5906 | 4.0373 | -2.2691 | 2.49E-02 | 2.83E-01 | -4.8079 |
| TINAGL1 | 0.5715 | 3.1008 | 2.2691 | 2.49E-02 | 2.83E-01 | -4.8079 |
| HSD17B6 | 0.9127 | 2.8082 | 2.2689 | 2.50E-02 | 2.83E-01 | -4.8084 |
| MTCO1P12 | -0.9595 | 6.1165 | -2.2671 | 2.51E-02 | 2.84E-01 | -4.8122 |
| MARCHF9 | 0.5018 | 3.2917 | 2.2649 | 2.52E-02 | 2.85E-01 | -4.8171 |
| HTRA1 | 0.5590 | 5.1219 | 2.2644 | 2.52E-02 | 2.85E-01 | -4.8181 |
| CAVIN1 | 0.5322 | 5.4975 | 2.2615 | 2.54E-02 | 2.86E-01 | -4.8244 |
| AC005077.14 | -0.6449 | 1.1227 | -2.2611 | 2.54E-02 | 2.86E-01 | -4.8253 |
| GAPDH | -0.5114 | 9.2394 | -2.2602 | 2.55E-02 | 2.86E-01 | -4.8274 |
| CCDC80 | 0.6149 | 2.9813 | 2.2600 | 2.55E-02 | 2.87E-01 | -4.8279 |
| AKR7A3 | -0.9633 | 1.7744 | -2.2599 | 2.55E-02 | 2.87E-01 | -4.8280 |
| FCGR3A | 0.6844 | 4.6464 | 2.2598 | 2.55E-02 | 2.87E-01 | -4.8281 |
| TAGLN | 0.5862 | 5.3112 | 2.2585 | 2.56E-02 | 2.87E-01 | -4.8311 |
| FCN3 | 0.8513 | 2.0639 | 2.2582 | 2.56E-02 | 2.87E-01 | -4.8316 |
| ZNF486 | 0.8320 | 2.2089 | 2.2573 | 2.57E-02 | 2.87E-01 | -4.8336 |
| HLA-DMA | 0.7127 | 5.9752 | 2.2559 | 2.58E-02 | 2.87E-01 | -4.8366 |
| GLB1L2 | 0.6552 | 2.5375 | 2.2537 | 2.59E-02 | 2.88E-01 | -4.8414 |
| SELENOP | 0.5879 | 3.7517 | 2.2533 | 2.59E-02 | 2.88E-01 | -4.8423 |
| SCD | -0.6552 | 6.0495 | -2.2449 | 2.65E-02 | 2.91E-01 | -4.8606 |
| CPLX2 | -0.9277 | 0.5110 | -2.2445 | 2.65E-02 | 2.91E-01 | -4.8615 |
| SLCO4C1 | 0.6492 | 1.6276 | 2.2426 | 2.66E-02 | 2.92E-01 | -4.8656 |
| IRF8 | 0.5622 | 2.4585 | 2.2422 | 2.67E-02 | 2.92E-01 | -4.8664 |
| IFI44 | 0.7987 | 4.0064 | 2.2413 | 2.67E-02 | 2.92E-01 | -4.8684 |
| MAGEB2 | -0.6510 | 0.3389 | -2.2388 | 2.69E-02 | 2.93E-01 | -4.8738 |
| ABHD11-AS1 | 0.6483 | 2.6571 | 2.2372 | 2.70E-02 | 2.94E-01 | -4.8773 |
| TMEM119 | 0.5866 | 3.2422 | 2.2339 | 2.72E-02 | 2.95E-01 | -4.8844 |
| CLTRN | 0.8698 | 1.8753 | 2.2326 | 2.73E-02 | 2.95E-01 | -4.8871 |
| ZNF90 | 0.5164 | 1.1046 | 2.2326 | 2.73E-02 | 2.95E-01 | -4.8872 |
| PPP1R1B | 1.1663 | 2.8758 | 2.2307 | 2.75E-02 | 2.96E-01 | -4.8913 |
| CHIT1 | 1.0408 | 2.6814 | 2.2291 | 2.76E-02 | 2.96E-01 | -4.8948 |
| RP11-499O7.7 | -0.8217 | 1.1311 | -2.2267 | 2.77E-02 | 2.97E-01 | -4.8999 |
| GALNT18 | 0.5142 | 3.1841 | 2.2259 | 2.78E-02 | 2.98E-01 | -4.9015 |
| TMEM98 | 0.5833 | 3.7440 | 2.2254 | 2.78E-02 | 2.98E-01 | -4.9028 |
| ALKAL1 | 0.5255 | 1.0192 | 2.2246 | 2.79E-02 | 2.98E-01 | -4.9045 |
| LTB | 0.6911 | 3.6391 | 2.2206 | 2.81E-02 | 2.99E-01 | -4.9130 |
| TNFSF13B | 0.5146 | 2.4505 | 2.2174 | 2.84E-02 | 3.00E-01 | -4.9199 |
| IER5L | -0.6141 | 2.8129 | -2.2171 | 2.84E-02 | 3.00E-01 | -4.9204 |
| PKDCC | 0.5660 | 2.0273 | 2.2155 | 2.85E-02 | 3.00E-01 | -4.9240 |
| APOC1 | 0.7919 | 5.8933 | 2.2147 | 2.86E-02 | 3.01E-01 | -4.9257 |
| RP11-818F20.5 | -0.5702 | 0.5384 | -2.2107 | 2.88E-02 | 3.02E-01 | -4.9343 |
| KCTD14 | 0.5569 | 1.8431 | 2.2081 | 2.90E-02 | 3.04E-01 | -4.9398 |
| CMPK2 | 0.5010 | 2.0785 | 2.2074 | 2.91E-02 | 3.04E-01 | -4.9413 |
| UGDH | -0.7748 | 4.8043 | -2.2070 | 2.91E-02 | 3.04E-01 | -4.9420 |
| GDF10 | 0.5756 | 0.9568 | 2.2052 | 2.92E-02 | 3.05E-01 | -4.9460 |
| GIMAP4 | 0.5335 | 3.6158 | 2.2032 | 2.94E-02 | 3.06E-01 | -4.9502 |
| CLIC5 | 0.7877 | 1.8268 | 2.2026 | 2.94E-02 | 3.06E-01 | -4.9515 |
| TNNC1 | 0.7475 | 2.4386 | 2.1955 | 2.99E-02 | 3.09E-01 | -4.9666 |
| OGN | 0.5329 | 1.0915 | 2.1953 | 3.00E-02 | 3.09E-01 | -4.9670 |
| TFPI2 | 1.4834 | 3.6900 | 2.1952 | 3.00E-02 | 3.09E-01 | -4.9672 |
| MBIP | 0.6116 | 4.4960 | 2.1917 | 3.02E-02 | 3.10E-01 | -4.9746 |
| NDRG2 | 0.5042 | 2.4256 | 2.1900 | 3.03E-02 | 3.10E-01 | -4.9782 |
| HSD11B1 | 0.5319 | 2.0541 | 2.1895 | 3.04E-02 | 3.10E-01 | -4.9793 |
| BMP2 | 0.7114 | 2.6034 | 2.1881 | 3.05E-02 | 3.11E-01 | -4.9822 |
| HPGD | 1.1378 | 3.4825 | 2.1876 | 3.05E-02 | 3.11E-01 | -4.9833 |
| HABP2 | 1.0563 | 2.4120 | 2.1850 | 3.07E-02 | 3.12E-01 | -4.9888 |
| TDRD5 | -0.5316 | 0.7294 | -2.1828 | 3.09E-02 | 3.13E-01 | -4.9936 |
| NDN | 0.5620 | 3.0965 | 2.1803 | 3.11E-02 | 3.14E-01 | -4.9987 |
| ENDOD1 | 0.5432 | 3.9884 | 2.1801 | 3.11E-02 | 3.14E-01 | -4.9992 |
| CRYM | 1.0232 | 2.3965 | 2.1791 | 3.12E-02 | 3.14E-01 | -5.0014 |
| CACNA1H | -0.6472 | 1.4095 | -2.1790 | 3.12E-02 | 3.14E-01 | -5.0014 |
| GALNT6 | 0.7739 | 3.1184 | 2.1789 | 3.12E-02 | 3.14E-01 | -5.0017 |
| GGT5 | 0.5427 | 3.0022 | 2.1786 | 3.12E-02 | 3.14E-01 | -5.0023 |
| CXCL12 | 0.5999 | 2.5003 | 2.1777 | 3.13E-02 | 3.15E-01 | -5.0042 |
| AKR1C2 | -1.7234 | 2.7010 | -2.1755 | 3.14E-02 | 3.16E-01 | -5.0089 |
| NUSAP1 | -0.5509 | 3.0960 | -2.1745 | 3.15E-02 | 3.16E-01 | -5.0109 |
| SDCBP2 | -0.8274 | 2.6866 | -2.1740 | 3.16E-02 | 3.16E-01 | -5.0120 |
| TEF | 0.5189 | 2.8164 | 2.1709 | 3.18E-02 | 3.17E-01 | -5.0186 |
| ZNF385B | 0.7546 | 1.4128 | 2.1706 | 3.18E-02 | 3.17E-01 | -5.0191 |
| GPSM3 | 0.5511 | 4.4325 | 2.1696 | 3.19E-02 | 3.18E-01 | -5.0211 |
| RP13-104F24.3 | 0.5615 | 3.9753 | 2.1690 | 3.19E-02 | 3.18E-01 | -5.0225 |
| PCOLCE2 | 0.5479 | 1.1630 | 2.1676 | 3.20E-02 | 3.19E-01 | -5.0254 |
| CNN1 | 0.5649 | 2.2614 | 2.1675 | 3.21E-02 | 3.19E-01 | -5.0256 |
| LL21NC02-1C16.2 | 0.7702 | 1.6429 | 2.1673 | 3.21E-02 | 3.19E-01 | -5.0260 |
| SFTA3 | 0.9855 | 4.7365 | 2.1665 | 3.21E-02 | 3.19E-01 | -5.0277 |
| SGK1 | 0.5122 | 3.3095 | 2.1625 | 3.24E-02 | 3.21E-01 | -5.0361 |
| C8orf34-AS1 | 0.7565 | 1.6675 | 2.1616 | 3.25E-02 | 3.21E-01 | -5.0379 |
| IRX6 | 0.6277 | 0.9300 | 2.1606 | 3.26E-02 | 3.21E-01 | -5.0400 |
| NCF2 | 0.5620 | 3.6643 | 2.1575 | 3.28E-02 | 3.22E-01 | -5.0465 |
| KRT19 | 0.7236 | 8.0429 | 2.1556 | 3.30E-02 | 3.23E-01 | -5.0506 |
| TPX2 | -0.7103 | 3.4095 | -2.1555 | 3.30E-02 | 3.23E-01 | -5.0508 |
| RP11-259K15.2 | 0.6998 | 1.5132 | 2.1484 | 3.36E-02 | 3.26E-01 | -5.0655 |
| CCL3 | 0.5306 | 2.3757 | 2.1484 | 3.36E-02 | 3.26E-01 | -5.0656 |
| SCUBE3 | 0.6818 | 1.4854 | 2.1483 | 3.36E-02 | 3.26E-01 | -5.0658 |
| RP11-760H22.2 | 0.5191 | 2.2536 | 2.1481 | 3.36E-02 | 3.26E-01 | -5.0661 |
| IGHA2 | 1.0954 | 7.5275 | 2.1465 | 3.37E-02 | 3.26E-01 | -5.0695 |
| CISH | 0.5557 | 3.3710 | 2.1464 | 3.37E-02 | 3.27E-01 | -5.0697 |
| SCUBE2 | 0.6812 | 1.5606 | 2.1434 | 3.40E-02 | 3.28E-01 | -5.0759 |
| MISP | -0.6577 | 4.5041 | -2.1419 | 3.41E-02 | 3.28E-01 | -5.0791 |
| SCG3 | -0.5247 | 0.3351 | -2.1405 | 3.42E-02 | 3.29E-01 | -5.0820 |
| KCNH2 | -0.6235 | 1.1362 | -2.1397 | 3.43E-02 | 3.29E-01 | -5.0836 |
| IGF2BP2 | 0.6497 | 2.2826 | 2.1391 | 3.43E-02 | 3.30E-01 | -5.0849 |
| CYP4F11 | -0.8693 | 1.2438 | -2.1369 | 3.45E-02 | 3.30E-01 | -5.0894 |
| CYP4B1 | 1.2551 | 3.8286 | 2.1348 | 3.47E-02 | 3.31E-01 | -5.0937 |
| KRT8P3 | -0.5045 | 1.3514 | -2.1345 | 3.47E-02 | 3.31E-01 | -5.0945 |
| SRXN1 | -0.5278 | 1.0839 | -2.1334 | 3.48E-02 | 3.31E-01 | -5.0968 |
| FILIP1L | 0.5258 | 2.9289 | 2.1333 | 3.48E-02 | 3.31E-01 | -5.0968 |
| LUCAT1 | -0.5986 | 1.0817 | -2.1313 | 3.50E-02 | 3.32E-01 | -5.1009 |
| HTR2B | 0.5561 | 0.8369 | 2.1310 | 3.50E-02 | 3.32E-01 | -5.1017 |
| IL27RA | 0.5271 | 3.7515 | 2.1288 | 3.52E-02 | 3.32E-01 | -5.1061 |
| GAPDHP1 | -0.8373 | 2.1646 | -2.1277 | 3.53E-02 | 3.33E-01 | -5.1085 |
| AMOT | 0.5933 | 1.8047 | 2.1263 | 3.54E-02 | 3.33E-01 | -5.1113 |
| CDCP1 | -0.5769 | 3.2735 | -2.1260 | 3.54E-02 | 3.33E-01 | -5.1119 |
| CLDN1 | 0.7877 | 3.9439 | 2.1255 | 3.55E-02 | 3.33E-01 | -5.1129 |
| ADAMTS8 | 0.5284 | 0.9911 | 2.1254 | 3.55E-02 | 3.33E-01 | -5.1131 |
| SHE | 0.6255 | 1.8502 | 2.1247 | 3.55E-02 | 3.34E-01 | -5.1146 |
| HLA-DQA2 | 1.1193 | 4.3150 | 2.1246 | 3.56E-02 | 3.34E-01 | -5.1149 |
| SPDEF | 0.8256 | 4.0422 | 2.1245 | 3.56E-02 | 3.34E-01 | -5.1150 |
| PRODH | 0.8188 | 2.2802 | 2.1229 | 3.57E-02 | 3.34E-01 | -5.1182 |
| OSMR | 0.5174 | 4.7284 | 2.1208 | 3.59E-02 | 3.35E-01 | -5.1226 |
| SELPLG | 0.5218 | 3.8523 | 2.1169 | 3.62E-02 | 3.36E-01 | -5.1307 |
| THBS4 | 0.7101 | 1.3061 | 2.1160 | 3.63E-02 | 3.36E-01 | -5.1325 |
| ACKR2 | 0.5105 | 0.7714 | 2.1145 | 3.64E-02 | 3.37E-01 | -5.1356 |
| ADRA2A | 0.7497 | 1.8901 | 2.1115 | 3.67E-02 | 3.38E-01 | -5.1418 |
| TUBA1A | 0.5263 | 5.1406 | 2.1105 | 3.68E-02 | 3.39E-01 | -5.1437 |
| ELF5 | 0.6900 | 1.3989 | 2.1103 | 3.68E-02 | 3.39E-01 | -5.1442 |
| PTGR1 | -0.6783 | 3.6901 | -2.1098 | 3.68E-02 | 3.39E-01 | -5.1451 |
| SLIT3 | 0.5196 | 1.4711 | 2.1087 | 3.69E-02 | 3.39E-01 | -5.1476 |
| EFEMP1 | 0.6316 | 4.3854 | 2.1061 | 3.72E-02 | 3.41E-01 | -5.1528 |
| ITGAX | 0.5125 | 3.0024 | 2.0996 | 3.77E-02 | 3.43E-01 | -5.1659 |
| RHOV | -1.0078 | 3.5436 | -2.0953 | 3.81E-02 | 3.45E-01 | -5.1747 |
| CTD-3128G10.7 | -0.5272 | 1.0104 | -2.0938 | 3.83E-02 | 3.46E-01 | -5.1778 |
| CD53 | 0.5683 | 4.7854 | 2.0902 | 3.86E-02 | 3.47E-01 | -5.1851 |
| BEX3 | -0.5372 | 5.9055 | -2.0896 | 3.86E-02 | 3.47E-01 | -5.1863 |
| IGHA1 | 1.0170 | 10.8597 | 2.0893 | 3.87E-02 | 3.47E-01 | -5.1869 |
| NPY | -0.6582 | 0.2564 | -2.0831 | 3.92E-02 | 3.50E-01 | -5.1994 |
| HOXD1 | 0.6823 | 1.4722 | 2.0827 | 3.93E-02 | 3.50E-01 | -5.2003 |
| SDC2 | 0.5067 | 3.2054 | 2.0796 | 3.96E-02 | 3.52E-01 | -5.2067 |
| KLHDC7A | 0.5511 | 1.5643 | 2.0783 | 3.97E-02 | 3.52E-01 | -5.2092 |
| ITGA3 | 0.6082 | 5.5531 | 2.0771 | 3.98E-02 | 3.53E-01 | -5.2116 |
| NQO1 | -1.0149 | 5.5722 | -2.0767 | 3.98E-02 | 3.53E-01 | -5.2123 |
| GLB1L3 | 0.8675 | 1.2916 | 2.0743 | 4.01E-02 | 3.54E-01 | -5.2173 |
| KIT | 0.8575 | 2.9272 | 2.0732 | 4.02E-02 | 3.54E-01 | -5.2195 |
| BMP3 | 0.8189 | 1.9568 | 2.0728 | 4.02E-02 | 3.54E-01 | -5.2203 |
| FOXQ1 | 0.9008 | 3.0628 | 2.0723 | 4.03E-02 | 3.55E-01 | -5.2213 |
| ACTA2 | 0.5451 | 5.6463 | 2.0722 | 4.03E-02 | 3.55E-01 | -5.2214 |
| ANPEP | 0.7170 | 2.7236 | 2.0720 | 4.03E-02 | 3.55E-01 | -5.2219 |
| AURKA | -0.5216 | 2.8020 | -2.0621 | 4.12E-02 | 3.59E-01 | -5.2418 |
| TRPC6 | 0.6614 | 1.8232 | 2.0585 | 4.16E-02 | 3.60E-01 | -5.2489 |
| RP11-206P5.2 | 0.5059 | 0.8765 | 2.0562 | 4.18E-02 | 3.61E-01 | -5.2535 |
| ALDH1A1 | 0.8543 | 4.6161 | 2.0554 | 4.19E-02 | 3.61E-01 | -5.2551 |
| CTD-2139B15.5 | -0.6567 | 0.6517 | -2.0526 | 4.22E-02 | 3.62E-01 | -5.2606 |
| RP11-284F21.7 | -0.6687 | 1.3614 | -2.0515 | 4.23E-02 | 3.63E-01 | -5.2630 |
| GATA6 | 0.5205 | 2.0579 | 2.0490 | 4.25E-02 | 3.64E-01 | -5.2679 |
| ATP10A | 0.5078 | 1.8900 | 2.0489 | 4.25E-02 | 3.64E-01 | -5.2681 |
| VEGFD | 0.7031 | 1.3578 | 2.0489 | 4.25E-02 | 3.64E-01 | -5.2682 |
| LINC00261 | 0.7564 | 1.4875 | 2.0485 | 4.26E-02 | 3.64E-01 | -5.2690 |
| IGHD3-16 | -0.5039 | 0.6775 | -2.0455 | 4.29E-02 | 3.65E-01 | -5.2750 |
| MAGEC2 | -0.6760 | 0.4481 | -2.0451 | 4.29E-02 | 3.65E-01 | -5.2756 |
| HSPB6 | 0.5837 | 2.0308 | 2.0445 | 4.30E-02 | 3.65E-01 | -5.2769 |
| CHST2 | 0.5496 | 2.2784 | 2.0413 | 4.33E-02 | 3.67E-01 | -5.2831 |
| PLEKHG4B | -0.5694 | 1.1406 | -2.0402 | 4.34E-02 | 3.67E-01 | -5.2853 |
| H2BC8 | -0.7724 | 1.5809 | -2.0390 | 4.35E-02 | 3.67E-01 | -5.2878 |
| RGCC | 0.5568 | 5.2456 | 2.0379 | 4.36E-02 | 3.68E-01 | -5.2900 |
| SEMA3A | 0.5445 | 1.2419 | 2.0354 | 4.39E-02 | 3.70E-01 | -5.2949 |
| ABCC2 | -0.8298 | 0.8626 | -2.0339 | 4.40E-02 | 3.70E-01 | -5.2978 |
| PTPRC | 0.5783 | 3.0712 | 2.0321 | 4.42E-02 | 3.71E-01 | -5.3015 |
| NPC2 | 0.6807 | 7.4915 | 2.0282 | 4.46E-02 | 3.73E-01 | -5.3092 |
| CD37 | 0.5265 | 3.2143 | 2.0275 | 4.47E-02 | 3.74E-01 | -5.3105 |
| ADGRG6 | 0.5143 | 2.2357 | 2.0274 | 4.47E-02 | 3.74E-01 | -5.3107 |
| CHML | -0.5378 | 2.5821 | -2.0231 | 4.52E-02 | 3.75E-01 | -5.3192 |
| PCDHB5 | 0.5181 | 1.0954 | 2.0209 | 4.54E-02 | 3.76E-01 | -5.3235 |
| CPXM2 | 0.5475 | 2.0520 | 2.0206 | 4.54E-02 | 3.76E-01 | -5.3240 |
| TMPRSS11E | -0.9964 | 1.8824 | -2.0200 | 4.55E-02 | 3.76E-01 | -5.3252 |
| FBN1 | 0.5185 | 2.6745 | 2.0196 | 4.55E-02 | 3.76E-01 | -5.3260 |
| ACTBP12 | -0.6022 | 0.3793 | -2.0160 | 4.59E-02 | 3.78E-01 | -5.3332 |
| AKR1C1 | -1.3499 | 2.6731 | -2.0128 | 4.62E-02 | 3.79E-01 | -5.3395 |
| FABP4 | 0.7676 | 1.6703 | 2.0114 | 4.64E-02 | 3.80E-01 | -5.3422 |
| GCLM | -0.5556 | 3.0300 | -2.0101 | 4.65E-02 | 3.80E-01 | -5.3446 |
| DUSP4 | -0.8629 | 3.0224 | -2.0095 | 4.66E-02 | 3.80E-01 | -5.3458 |
| CEACAM6 | 1.0069 | 8.3629 | 2.0082 | 4.67E-02 | 3.81E-01 | -5.3484 |
| PLAC8 | 0.6279 | 1.9969 | 2.0074 | 4.68E-02 | 3.81E-01 | -5.3500 |
| P3H3 | 0.5225 | 2.5933 | 2.0066 | 4.69E-02 | 3.81E-01 | -5.3515 |
| SLC7A5 | -0.7285 | 4.4721 | -2.0054 | 4.70E-02 | 3.82E-01 | -5.3539 |
| ME1 | -0.5494 | 3.1196 | -2.0038 | 4.72E-02 | 3.82E-01 | -5.3569 |
| C4B | 0.5363 | 1.8739 | 2.0035 | 4.72E-02 | 3.82E-01 | -5.3576 |
| AGER | 1.1332 | 4.2196 | 2.0032 | 4.73E-02 | 3.83E-01 | -5.3582 |
| RP11-510N19.5 | -0.5369 | 2.5450 | -2.0026 | 4.73E-02 | 3.83E-01 | -5.3594 |
| STX1A | -0.5646 | 2.5719 | -2.0024 | 4.74E-02 | 3.83E-01 | -5.3598 |
| DPP10-AS1 | -0.6199 | 1.0437 | -1.9971 | 4.79E-02 | 3.85E-01 | -5.3701 |
| CDH11 | 0.5069 | 2.8335 | 1.9970 | 4.80E-02 | 3.85E-01 | -5.3702 |
| LYNX1 | 0.5368 | 1.6786 | 1.9937 | 4.83E-02 | 3.87E-01 | -5.3766 |
| IFI6 | 0.7830 | 7.3630 | 1.9929 | 4.84E-02 | 3.87E-01 | -5.3782 |
| NMB | -0.5235 | 3.1277 | -1.9910 | 4.86E-02 | 3.88E-01 | -5.3818 |
| CCL18 | 0.9284 | 5.4331 | 1.9894 | 4.88E-02 | 3.88E-01 | -5.3850 |
| CRISPLD1 | 0.6114 | 1.4165 | 1.9887 | 4.89E-02 | 3.88E-01 | -5.3864 |
| VIPR1 | 0.5004 | 1.7498 | 1.9885 | 4.89E-02 | 3.88E-01 | -5.3867 |
| COX4I2 | 0.5287 | 2.6068 | 1.9881 | 4.89E-02 | 3.88E-01 | -5.3874 |
| UBXN10 | 0.6030 | 2.1024 | 1.9881 | 4.89E-02 | 3.88E-01 | -5.3876 |
| CAV2 | 0.5723 | 3.2107 | 1.9873 | 4.90E-02 | 3.89E-01 | -5.3890 |
| H1-12P | -0.6091 | 1.0806 | -1.9853 | 4.93E-02 | 3.90E-01 | -5.3929 |
| MAGEA12 | -0.7684 | 0.6235 | -1.9809 | 4.98E-02 | 3.92E-01 | -5.4014 |
| FADS2 | -0.6312 | 3.0865 | -1.9809 | 4.98E-02 | 3.92E-01 | -5.4014 |
| HOPX | 0.8527 | 4.5512 | 1.9807 | 4.98E-02 | 3.92E-01 | -5.4017 |
| HLA-G | 0.7288 | 2.4045 | 1.9802 | 4.98E-02 | 3.92E-01 | -5.4028 |
| B3GNT7 | 0.8102 | 4.2195 | 1.9802 | 4.98E-02 | 3.92E-01 | -5.4028 |
| MX2 | 0.5047 | 2.3891 | 1.9800 | 4.99E-02 | 3.92E-01 | -5.4031 |
| VWA2 | 0.5525 | 2.5050 | 1.9792 | 4.99E-02 | 3.92E-01 | -5.4046 |
